# Sulfonamide derivatives as tubulin polymerization inhibitors: advances in structural mechanisms, structure–activity relationships, and therapeutic potential in cancer treatment

**DOI:** 10.1039/d5ra09801g

**Published:** 2026-03-31

**Authors:** Rohini Gupta, Iqubal Singh, Vijay Luxami, Kamaldeep Paul

**Affiliations:** a University Center for Research and Development, Chandigarh University Mohali India; b School of Pharmaceutical Sciences, Lovely Professional University Phagwara India; c Department of Chemistry and Biochemistry, Thapar Institute of Engineering and Technology Patiala-147001 India kpaul@thapar.edu

## Abstract

Cancer has been ranked as one of the major causes of death globally, with a rising trend in its incidence and resistance towards existing chemotherapeutic drugs. These necessitate the development of novel therapeutic strategies for the treatment of this deadly disease. Microtubules, which are integral to cell division, are potential target sites in drug discovery for anticancer drugs. Sulfonamide derivatives have emerged as potential and privileged scaffolds in medicinal chemistry, exhibiting significant potential in inhibiting tubulin polymerization due to their high binding affinity at the colchicine binding site of tubulin. Binding to the colchicine site rapidly induces G2/M-phase cell cycle arrest and ultimately cell death in rapidly proliferating cancer cells. The past decade has seen significant advancements in academia and the pharmaceutical industry in the development of sulfonamide-based anticancer agents including natural product hybrids, synthetic analogues and molecular conjugates, which have shown activity in multidrug-resistant cancer cells while exhibiting favorable pharmacokinetics. This review summarizes recent insights into structural mechanisms and structure–activity relationship (SAR) data, along with the latest preclinical and clinical advances. Several promising compounds that show potential to overcome the limitations of current microtubule-targeting agents are highlighted. Collectively, the evidences position sulfonamide derivatives as robust scaffolds for the development of next-generation cancer therapeutics targeting tubulin polymerization.

## Abstract 2

Sulfonamide derivatives are potential tubulin binding agents which specifically binds to the colchicine site of microtubule. Sulfonamide derivatives potentially disrupt the polymerization process of tubulin network and induce the cancer cell death.

## Graphical abstract

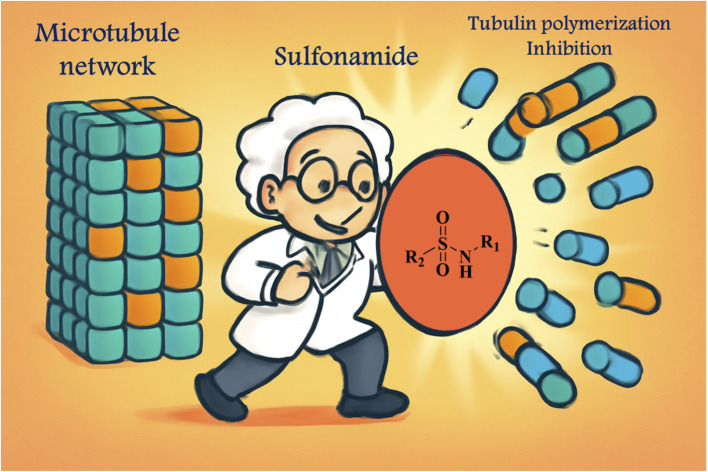

## 1. Introduction

Microtubules along with actin microfilaments and intermediate filaments are essential cytoskeleton components in eukaryotic cells. Microtubules are also essential for many important cellular functions such as cytokinesis, chromosome migration during mitosis, cell plate formation, organelle transport, the preservation of cellular architecture and regulation of mitotic spindle formation during cell division.^1^ Tubulin is a heterodimer of two globular subunits called α- and β-tubulin, and each of these are composed of approximately 450 amino acid residues.^2^ These two subunits have 40% amino acid sequence similarity. The polymerization of microtubules is initiated by the nucleation of a short protofilament seed made up of α,β-tubulin heterodimers. A rapid elongation phase ensues after the nucleation step and is driven by the noncovalent, head-to-tail addition of α,β-heterodimers to the growing microtubule ends.^3^ Starting with the hydrolysis of GTP coupled to the exchangeable nucleotide site of β-tubulin in the polymerization process, they laterally associate to produce a hollow microtubule. Any interference that leads to a disturbance in the microtubule dynamics results in mitotic arrest, which ultimately leads to cell death *via* apoptosis.^4^

### 1.1. Microtubulin binding sites and dynamics

**Fig. 1:**
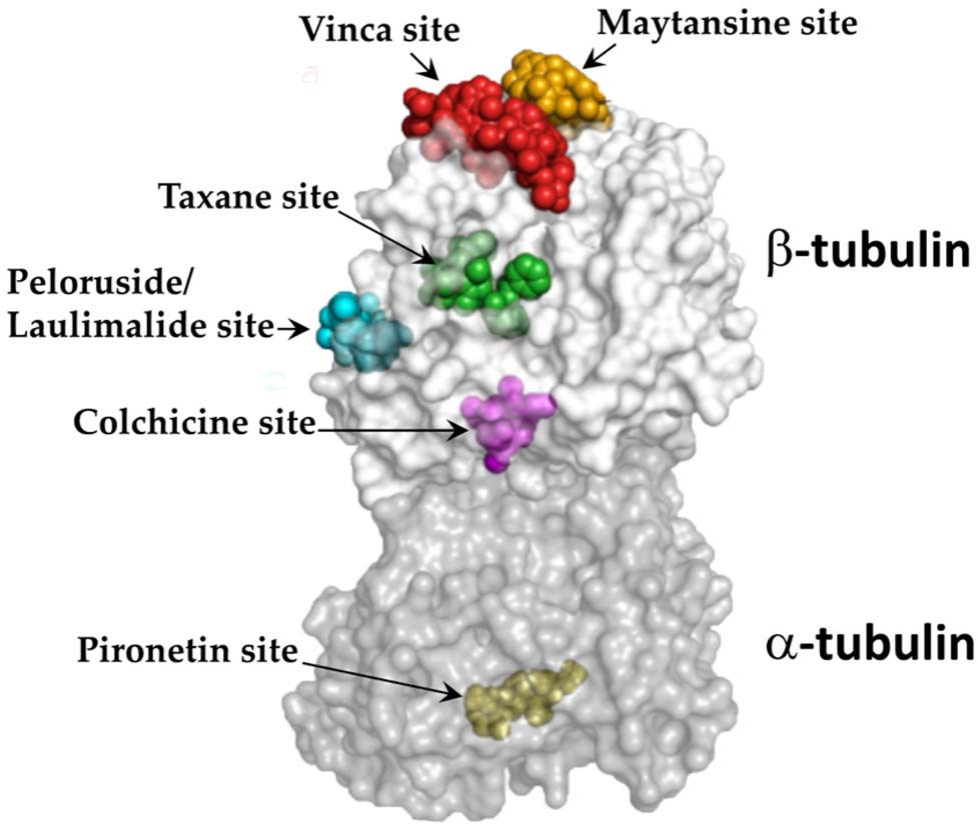
Microtubule-targeting agent (MTA) binding sites on tubulin. The α,β-tubulin dimer with bound ligands. To highlight the distinct binding sites, all ligands are shown in sphere representation and are colored in green (taxane site), cyan (laulimalide/peloruside site), magenta (colchicine site), slate (vinca site), orange (maytansine site), and yellow (pironetin site). Adapted with permission from Elsevier (ref. 9). Copyright 2018.

Microtubules are the largest and most rigid among the filamentous cytoskeletons, with an outer diameter of 25 nm.^5^ Structural studies have established several tubulin ligand-binding pockets such as taxane, vinca alkaloid, and colchicine sites.^6^ These pockets are bound by the microtubule-associated proteins (MAPs) and microtubule-targeting agents (MTAs). These locations serve as critical control centers, with therapeutic compounds influencing the behavior of microtubules by stabilizing or destabilizing the formation of polymers. To date, there are eight well-known binding sites on tubulin, namely, colchicine, gatorbulin, laulimalide, maytansine, taxane, pironetin, todalam, and vinca site.^7^ Recently, a new binding site known as tambulin site was also identified (Fig. 1).^8^ Different tubulin sites, their positions and functions are summarized in Table 1.

**Table 1:** Distinct tubulin binding sites, their positions and functions

Binding site	Position	Function
Colchicine site	Present in the middle domain of β-tubulin unit as a deep pocket	Microtubule formation inhibition
Gatorbulin site	Present between α,β-tubulins at their intradimer interface	Acts as microtubule destabilizing agent by interfering with structural assembly of the tubulin dimer, essential for normal polymerization
Maytansine	Present on the exposed β-tubulin pocket	Inhibit microtubule polymerization
Vinca site	Present on β-tubulin at the inter-dimer interface between two aligned tubulin dimers	Vinca site ligands interfere with the polymerization of microtubules, leading to the disruption of mitotic spindle formation and halting the cell division process
Pironetin site	Present on α-tubulin unit, near the N-terminal domain	Pironetin site ligand interacts with a specific cysteine residue (Cys316) on α-tubulin, leading to disrupted polymerization of tubulin and impaired mitotic spindle formation, ultimately causing cell cycle arrest
Todalam site	Present between the two tubulin dimers, binding to both tubulin dimers	Destabilizes microtubule network, causing cell death *via* cell cycle arrest in G2/M phase
Tumbulin site	Present at the interface of α1-tubulin, β1-tubulin, and stathmin-like protein B3 (RB3)	Enhances tubulin-depolymerizing activity of RB3
Laulimalide site	Present on β-tubulin, lies near the adjacent interfaces of protofilaments on the outer microtubule surface	Microtubule-stabilizing agent, promoting the stabilization of lateral interactions between adjacent protofilaments
Taxane site	Present in a pocket of β-tubulin, on the luminal side of microtubules	Inhibits mitosis, reduces dynamic instability, stabilizes microtubule network and promotes excessive and stable microtubule polymerization

### 1.2. Microtubule-targeting agents

Microtubule-targeting agents (MTAs) are a keystone of modern-day anticancer therapy. Due to their ability to disrupt the normal functioning of microtubules, MTAs have emerged as potential candidates for halting the cell cycle progression and inducing cell death.^10^ Hence, they are useful in a wide range of malignancies. MTAs have been classified into two categories based on their mechanism of action.^11^ The first category is microtubule-destabilizing agents (MDAs), which inhibit polymerization and promote depolymerization, *e.g.* alkaloids and colchicine. The second category is microtubule-stabilizing agents (MSAs), which stabilize microtubules and promote polymerization, *e.g.* taxanes and laulimalide/peloruside-A. Despite their different molecular mechanisms, both categories induce abnormality in microtubules and ultimately cell death.

**Fig. 2:**
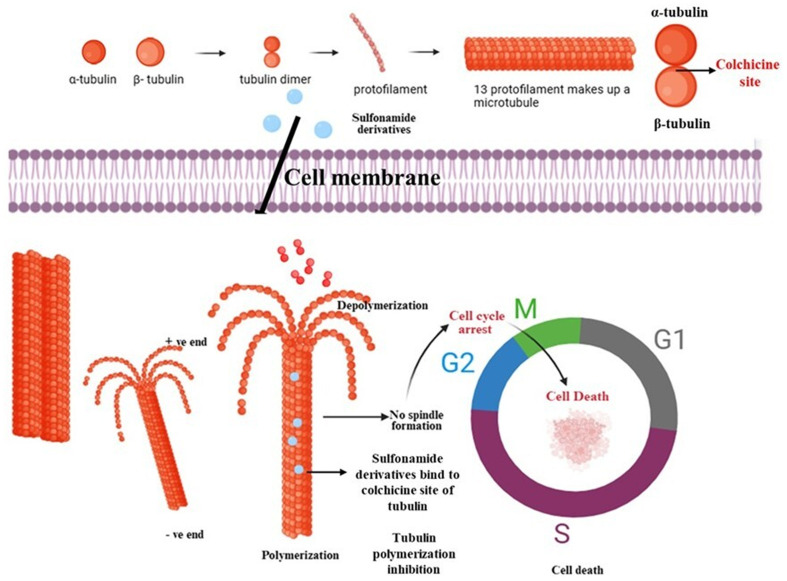
General schematic of the mechanism of action of sulfonamide derivatives targeting tubulin polymerization inhibition for cancer cell death.

Over the past several years, numerous reports have demonstrated that small molecules can effectively target tubulin by binding to specific sites on the α,β-tubulin heterodimers, such as the colchicine-binding domain, the vinca alkaloid site, and others. Microtubule polymerization inhibitors block the incorporation of α,β-tubulin heterodimers at the rapidly growing end, *i.e.* plus end, and simultaneously initiate their dissociation from the minus end. This process ultimately leads to microtubule destabilization and polymerization inhibition^12^ (Fig. 2). In contrast, microtubule-stabilizing agents promote the polymerization and suppress the depolymerization of microtubules by enhancing their stability and inducing cell death by mitotic arrest.^13^ Some anticancer therapeutic agents strategically exploit the essential requirement of dynamic tubulin exchange during mitosis by disrupting the well-balanced equilibrium between polymerization and depolymerization processes.^14^ Some drugs directly bind to the free tubulin heterodimers and prevent their further integration into the growing microtubule ends. In both cases, disruption of the microtubule dynamics prevents proper spindle formation, activates cell cycle check points and induces apoptosis.^15^

The first microtubule-targeting agents discovered were natural products such as colchicine (first identified tubulin inhibitor), vinca alkaloids and taxanes (paclitaxel-microtubule stabilizers).^16^ Vinca alkaloids and taxanes have become the foundation of chemotherapy in leukemias, lymphomas, and breast, lung, and ovarian cancers. Their success indicated that tubulin and microtubule dynamics were potential target sites for the treatment of cancer. However, clinical studies have revealed several challenges, and their use has been limited due to numerous drawbacks, such as poor pharmacokinetic profiles and lack of oral bioavailability. They also exhibit dose-limiting toxicities such as peripheral neuropathy, myelosuppression, and the development of multidrug resistance mediated by efflux pumps such as P-glycoprotein.^17^ This demonstrates the urgent need for the development of novel MTAs with improved specificity and lower resistance potential.^18^

In recent years, significant advancements such as crystallography and cryo-EM have revealed the structure and binding sites on tubulin.^19^ These structural insights provide a potential strategy to address the issues associated with conventional MTAs such as resistance and toxicity. These efforts led to the development of epothilones (*e.g.*, ixabepilone, approved in 2007), which showed superior activity against taxane-resistant tumors due to their ability to evade efflux pumps. In parallel, attention shifted to the colchicine-binding site, where small-molecule inhibitors such as combretastatins, chalcones, and sulfonamide derivatives emerged as vascular-disrupting agents with promising preclinical and clinical potential.^20^ To overcome the limitations, researchers also focused on the development of synthetic and semi-synthetic derivatives of taxanes, vinca alkaloids and epothilone but their dose-limiting toxicity still remains a major barrier. Structural insights into tubulin binding sites have enabled the rational design of novel small molecules to selectively target the colchicine site or novel binding domains for enhanced efficacy and reduced toxicity for potential activity against resistant tumors (Table 2). However, despite the significant advances, the clinical translation of many tubulin inhibitors, including sulfonamide derivatives, has been hindered by their suboptimal pharmacokinetic (PK) properties. These derivatives often suffer from poor aqueous solubility, low tumor penetration, low oral bioavailability and short half-life. These issues lead to challenges in achieving required therapeutic plasma concentrations and limit their clinical application. The other significant hindrance is the vulnerability to drug efflux through ATP-binding cassette transporters such as P-glycoprotein, which is also associated with multidrug resistance in cancer treatment.^21^ To overcome these constraints, a number of strategies are being explored. The use of medicinal chemistry strategies, such as structural changes to optimize the lipophilic–hydrophilic balance, prodrug development, *etc.*, has demonstrated potential in increasing the absorption and distribution of developed drug candidates with reduced toxicity profiles. Nanoparticle-based delivery systems, liposomal encapsulation and polymer–drug conjugates are also formulation strategies being developed to enhance the circulation time and tumor selectivity and reduce toxicity.^22^ Thus, researchers have focused on the rational design of new-generation sulfonamide derivatives with better metabolic stability and less efflux liability.^23^ All these efforts have the potential to alleviate the PK bottlenecks and facilitate the clinical development of sulfonamide-conjugated binders of tubulins. Beyond cancer, tubulin inhibitors are also being investigated for the treatment of infectious diseases, inflammatory disorders, and neurodegenerative conditions, highlighting their expanding clinical relevance. For example, Plinabulin, a synthetic analog with vascular disrupting and tubulin inhibition potential, is currently in late-phase clinical trials for non-small cell lung cancer and as a supportive care agent to reduce chemotherapy-induced neutropenia.^24^

**Table 2:** Some drug candidates in clinical trials and in market that show tubulin polymerization inhibition

S. no.	Drug candidate	Target site	MTD	IC_50_ anticancer activity	IC_50_ tubulin inhibition	Ref.
1	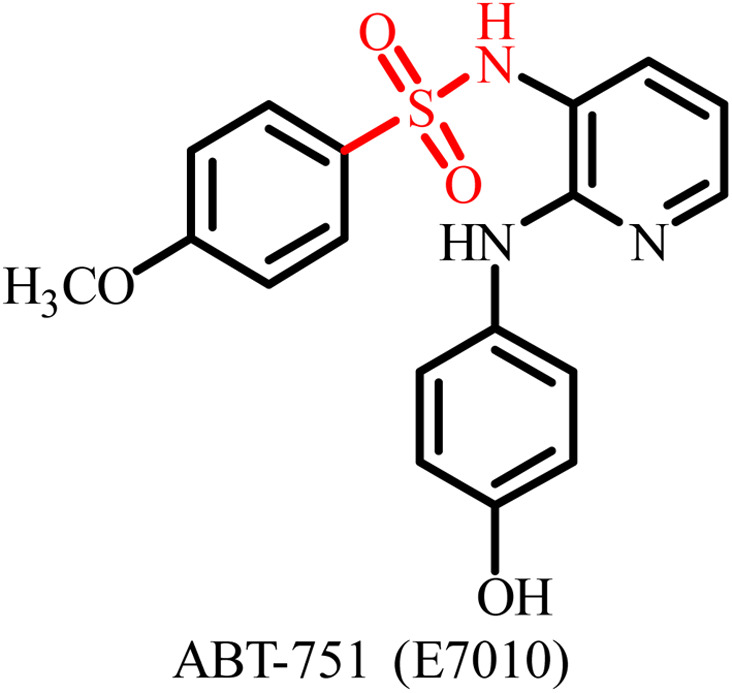	Inhibits tubulin polymerization and induces G2/M-phase cell cycle arrest	Fatigue, hematologic and gastrointestinal toxicities	208.2–1007.2 nM	3300 nM	25
2	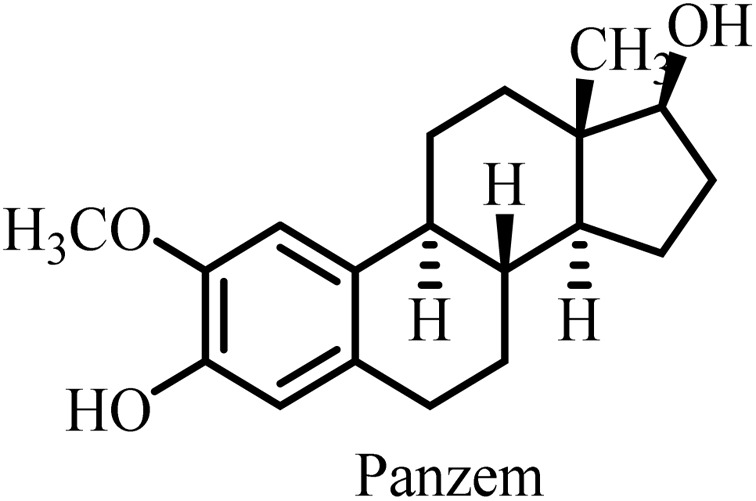	Microtubule destabilizer, causing cell cycle arrest during metaphase and cancer cell death	Fatigue and hypophosphatemia	1000–20000 nM	1200 nM	26
3	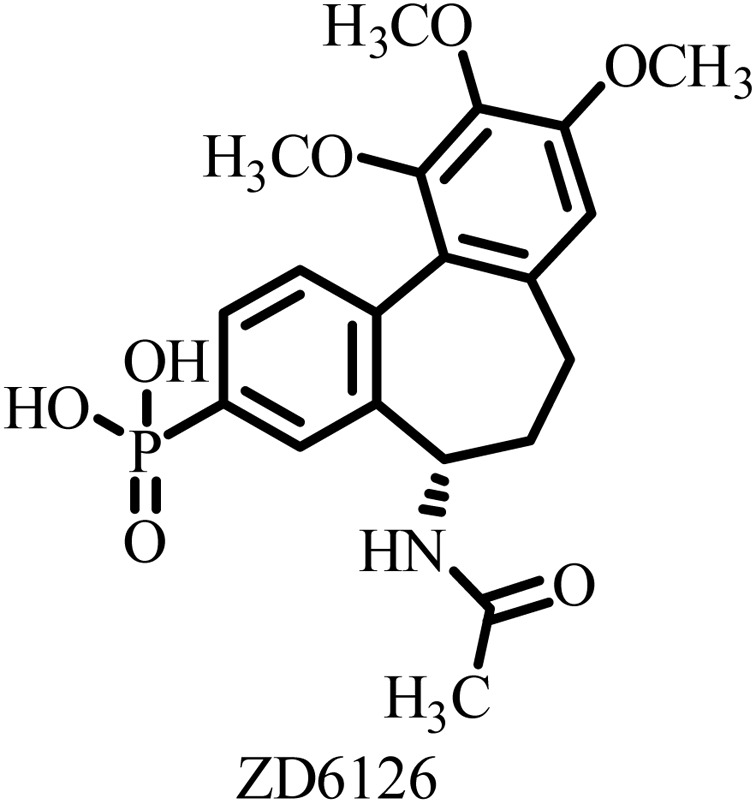	Tubulin binding agent, binds to colchicine site and inhibits tubulin polymerization	Hypoxia	10^4^–10^5^ µM	70–620 nM	27
4	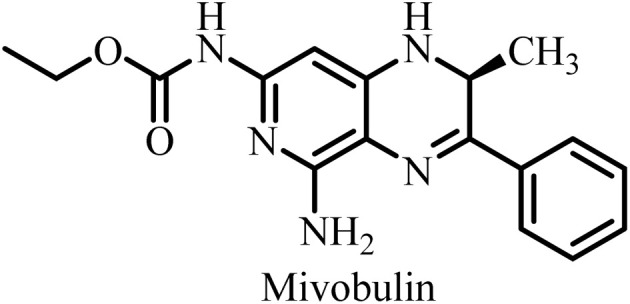	Inhibits microtubule polymerization	Neutropenia	N.A.	N.A.	28
5	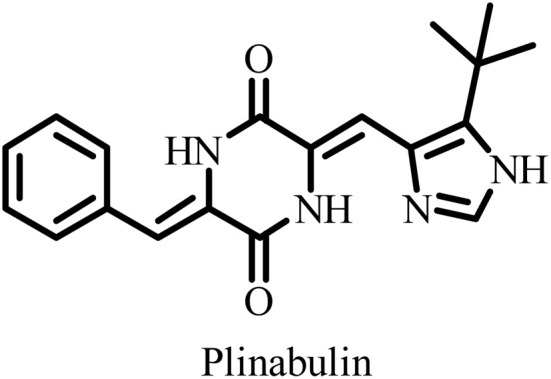	Inhibits tubulin polymerization and triggers vascular disruption	Grade 4 hematologic toxicity, grade 3 nonhematologic toxicity	8–10 nM	2400 nM	29
6	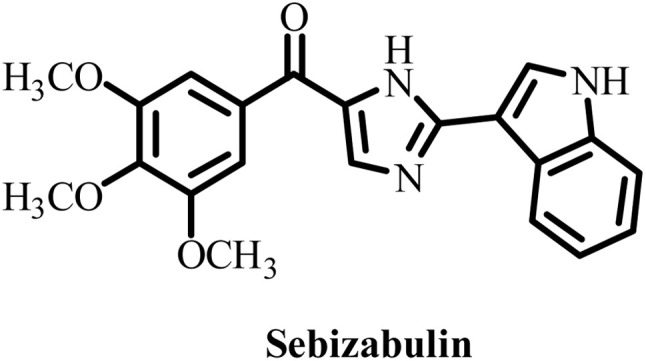	Oral colchicine-site binder and tubulin polymerization inhibitor	No DLT observed	9–10 nM	N.A.	30
7	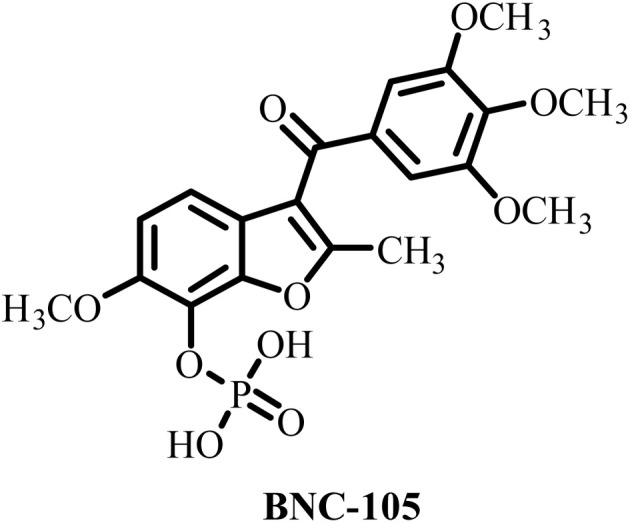	Vascular disrupting and tubulin polymerization inhibition	Grade 4 thrombocytopenia, grade 4 neutropenia	<1 nM	3000 nM	31
8	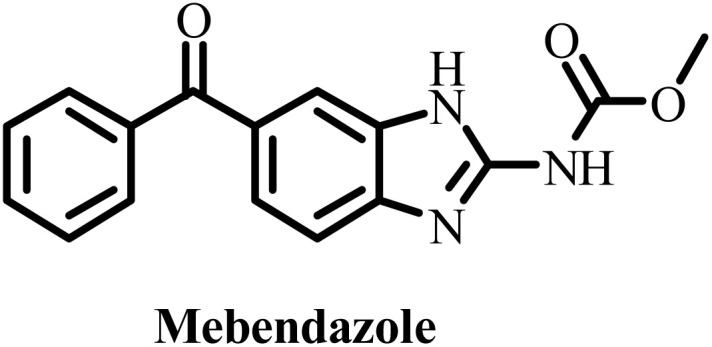	Inhibits tubulin polymerization	None	100–1250 nM	110–320 nM	32
9	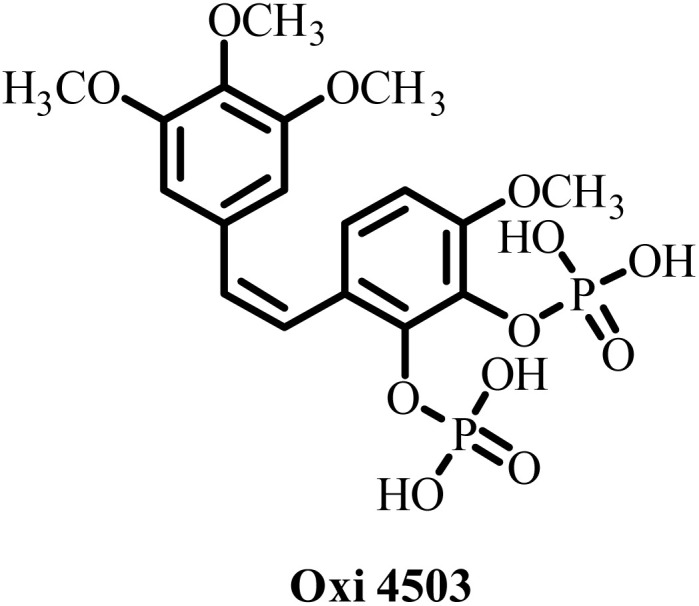	Dual-function VDA/tubulin depolymerizer	Atrial fibrillation, bowel perforation, blurred vision, diplopia and grade III increased troponin levels	NA	NA	33
10	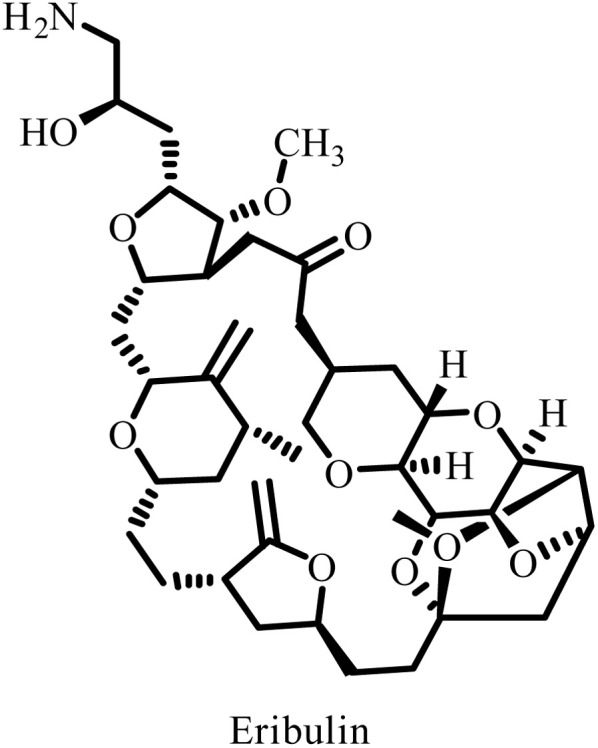	Microtubule disrupter, causing mitotic arrest, spindle failure and cancer cell death	Febrile neutropenia	0.09–9.5 nM	190 nM	34

### 1.3. Potential interactions essential for the efficient binding of MTAs with tubulin

CYS 241 is an important amino acid residue located on the S8 strand of the β-subunit in the colchicine binding site (CBS). CBS is a major drug target for destabilizing the microtubule and inhibiting polymerization.^35^ The major interactions of this residue are hydrophobic interactions, van der Waals interactions and H-bonding with ligands, which play a critical role in inhibition of polymerization, causing cell cycle arrest and apoptosis. It favors the binding of CBIs by serving as crucial targeting unit and H-bond acceptor for ligands. It occupies the central site of the hydrophobic pocket, binding with the ligand in the deep pocket and blocking conformational changes essential for polymerization.

Leu248, Ala250 and Asn258 are also critical amino acids located in the tubulin β-subunit of the colchicine binding site (CBS).^36,37^ These amino acids play an important role in stabilizing the interaction between tubulin and antimitotic agents. Their interactions lead to the inhibition of tubulin polymerization, causing cell cycle arrest in the G2/M phase and ultimately cancer cell death. The major interactions formed by CBIs with Leu248 and Ala250 are hydrophobic interactions and hydrogen bonding, thus enhancing the binding stability of the ligand within the hydrophobic pocket.

The Lys254 residue, particularly of the β-subunit plays a critical role in the efficient binding of small CBIs in the hydrophobic pocket of tubulin.^36,37^ It stabilizes the ligand and tubulin complexation mainly by H-bond and hydrophobic interactions. Several reports claimed that Lys254 serves as key residue for the stabilization of tubulin and well-known CBIs such as CA 84. Thus, it plays a key role in the enhanced binding affinity and stability of CBIs on the colchicine site of tubulin.

Structural and computational studies confirmed the presence of a Met259 amino acid residue in the CBS and its contribution to the binding of the CBI ligand to the colchicine site mainly *via* hydrophobic contacts. This was also observed in a docking study of the colchicine site of tubulin with the representative ligand colchicine.

Thr179 and Val181 are other important amino acid residues of the T5 loop of tubulin. These amino acids provide strong anchoring points for ligands to form hydrogen bonds and van der Waals interactions and stable binding of ligands at the αβ interface of the colchicine binding site.^38^

### 1.4. Recent developments in MTAs

Conventional MTAs, *e.g.* taxanes and vinca alkaloids, have shown remarkable clinical utility in the tumors of breast, ovarian, and lung cancers. However, these MTAs have not been able to fully eradicate these cancers due to drug resistance, neurotoxicity and other systemic toxicities, posing a major challenge for researchers worldwide. Thus, new strategies have become a top priority for the design and development of MTAs with better efficacy and safety profiles to manage toxicity. For example, optimized dosing schedules, formulation innovations, and combination therapies allow dose reduction without compromising efficacy, as well as the design of MTAs with selective binding profiles that spare neuronal microtubules. Advancements in structural biology have also significantly contributed to the development of new MTAs with enhanced therapeutic effects by providing a better understanding of their mechanism of action. For example, combretastatin A-4 (CA-4) isolated from the African tree *Combretum caffrum* is a well-known member of the combretastatin family and a potential antimitotic agent, which entered phase II and phase III clinical trials.^39^ However, it has several serious limitations such as low water solubility, short plasma half-life and ability to isomerize from active *cis*-isomeric form to inactive *trans*-isomeric form. To address these limitations, researchers developed fosbretabulin (CA-4P), a derivative of CA-4 with improved pharmacokinetic properties, which was approved by the FDA in 2018 and is used to treat thyroid cancer.^40^ After this clinical success, numerous salts of fosbretabulin and new derivatives have been developed, such as Oxi4503 and ombrabulin, which have been trialed as monotherapy and in combination therapy with well-known anticancer agents.^41^ Sabizabulin, another CBI with significant preclinical results, is also in clinical trials.^42^ These positive outcomes from preclinical and early clinical trials are encouraging for the development of next-generation MTAs.

### 1.5. Microtubule mutations

Drug resistance in cancer cells is caused by expression alterations, direct tubulin mutations, or isotype switching in the β-tubulin subunit of microtubules. For example, substitution of a serine amino acid residue with arginine at position 277 in β-tubulin causes steric repulsion between Arg277 and the adjacent Arg278 residue. This interaction causes alterations in the conformation of the tubulin M-loop, leading to the failed binding of paclitaxel and ultimately resistance to it. Mutations at positions A248T and M257V in the β-tubulin subunit cause resistance to colchicine-class drugs by reducing the binding energy by approximately 2-fold.^43^

The L240I mutation found near the colchicine binding site led to resistance to the well-known MT destabilizing anticancer drug vinblastine and colchicine by increasing MT stability.^44^ The L240I mutation is caused by substitution of leucine with isoleucine at a position close to Ala248, which may have resulted in a conformational change within the H7 helix.^45,46^ These mutations interfere with the longitudinal interaction between two monomers of tubulin, resulting in a change in their conformation. Alternatively, the L240F mutation led to resistance to ABT-751 and rigosertib.^47^

The presence of D197 and K350N mutations in cancer cells resulted in resistance against 2-methoxyestradiol, colchicine and vinca alkaloids. This study suggested that mutations play a significant role in resistance but not in drug binding ability. D197 present at the end of S6 contributes to the formation of a series of hydrogen bonds and none of the hydrogen bonds are disrupted by the mutation of the D197 amino acid residue. However, owing to the ability of D197 to participate in salt-bridge formation with R156 in the H4 helix, its removal results in the loss of H4 anchoring. This further causes alterations in the conformation within β-tubulin, leading to MT stability.^46^

### 1.6. Impact on drug discovery

The identification of these mutations has led to three significant shifts in pharmacological development.

(1) Unlike conventional MTAs such as taxanes (*e.g.* paclitaxel), CBIs are not the substrates for the P-glycoprotein (P-gp) efflux pump. Drug discovery and development technology is now shifting to the development of new and more efficient CBIs specifically to treat multidrug-resistant (MDR) tumors that have failed the standard-of-care taxane therapy.

(2) Upregulation of the βIII-tubulin isotype in cancer cells helps in the development of resistance to conventional MTA-based anticancer drugs. Modern drug design and discovery technology techniques are used to develop isotype-selective CBIs that bind specifically to the βIII isotype. This is a potential strategy to turn the resistance mechanism of cancer cells into a therapeutic target.^46^

(3) Recent studies show that targeting the hinge region of tubulin at the intermediate or transition state is a more effective strategy than targeting its pocket. Tubulin generally undergoes conformational changes from curved to straight, which are essential for microtubule polymerization and the survival of cancer cells. Targeting tubulin in the intermediate state is an effective strategy as drug candidates that target the interface of the tubulin dimer or hinge region prevent polymerization. This enables the developed drug candidates to address the mutations from which most of the CBIs suffer. Targeting the hinge region inhibits the curved-to-straight conformational switch and ultimately prevents the microtubule polymerization process. Todalam is a recently studied drug that acts as a molecular plug, inhibiting the curved to straight conformation of tubulin necessary for the polymerization process and preventing the development of resistance.

## 2. Sulfonamides as microtubule-targeting agents

Sulfonamides are amides of sulfonic acids, having the general formula of RSO_2_NR^1^R^2^, and depending on the number of substituents, they are classified as primary, secondary and tertiary sulfonamides. The sulfonamide functional group is considered a magic moiety and a privileged functional group by medicinal chemists. Thus, it is introduced as the main core for different bioactivities in drug candidates by various pharmaceutical industries and research centers. The discovery of the sulfonamide-based drug Prontosil as an effective antibacterial drug in the 1930s led to the development of a class of synthetic compounds (–SO_2_–NH– linkages) quickly and gave rise to many “sulfa” drugs (*e.g.* sulfamethoxazole) that inhibit bacterial dihydropteroate synthase.^48^ Many sulfonamides drugs are available on the market for the treatment of different cancers (Table 3). Sulfonamides have been described as molecular chimeras capable of forming hydrogen bonds and interacting within diverse environments. Their favourable properties, including accessibility, hydrogen-bonding ability, stability, hydrophobic–hydrophilic balance, polarity, and conformational flexibility, have driven significant interest in exploring sulfonamide chemistry. Thus, they have been incorporated into drug-like molecules to treat a variety of illnesses. Secondary and tertiary sulfonamides, in particular, have emerged as promising multiple kinase as well as tubulin polymerization inhibitors by binding to different sites on tubulin.^49^

**Table 3:** Currently available sulfonamide drugs with different target sites for the treatment of cancer

S. no.	Drug	Target site	DLT	IC_50_	Ref.
1	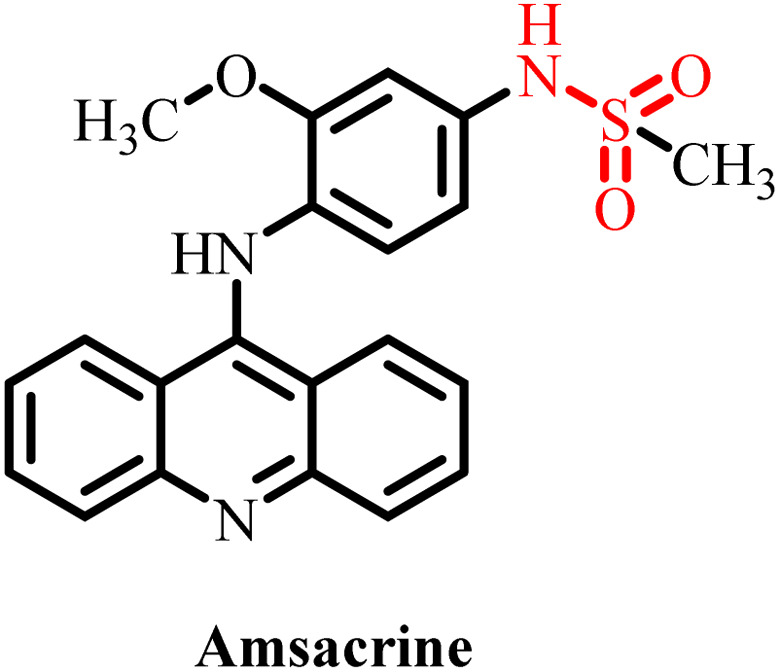	Topoisomerase II	Bone marrow suppression, hematological toxicity, gastrointestinal side effects, nausea, vomiting, stomatitis, diarrhea, and abdominal pain	1000–6000 nM	50
2	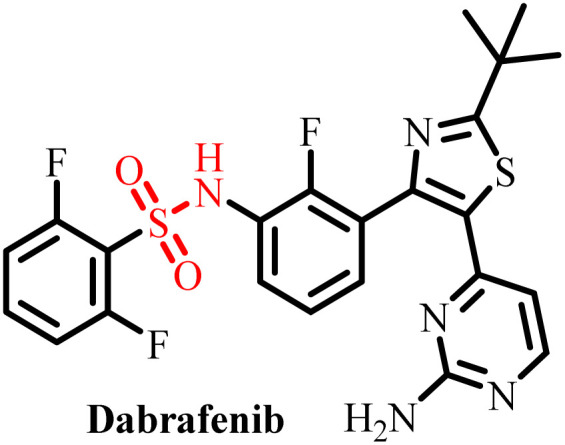	Kinase inhibitor	Grade 3 myelosuppression, grade 2 fatigue	0.65 nM	51
3	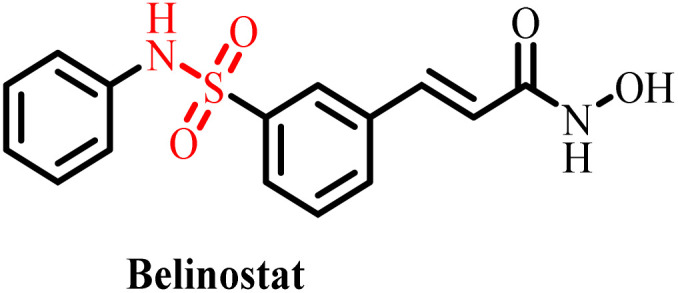	Pan-histone deacetylase (HDAC) inhibitor	Nausea/vomiting, diarrhea associated with fatigue, and atrial fibrillation	200–2000 nM	52
4	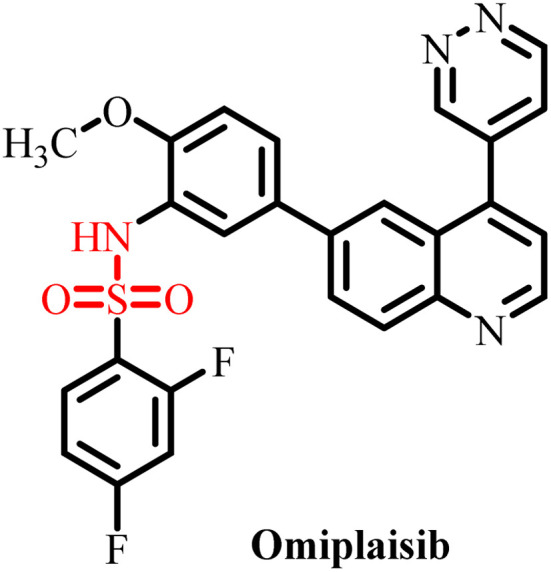	Inhibitor of phosphoinositide 3-kinase (PI3K) and the mammalian target of rapamycin (mTOR)	Diarrhea and skin rash	17.45 nM (OCI-AML3), 8.93 (THP-1)	53
5	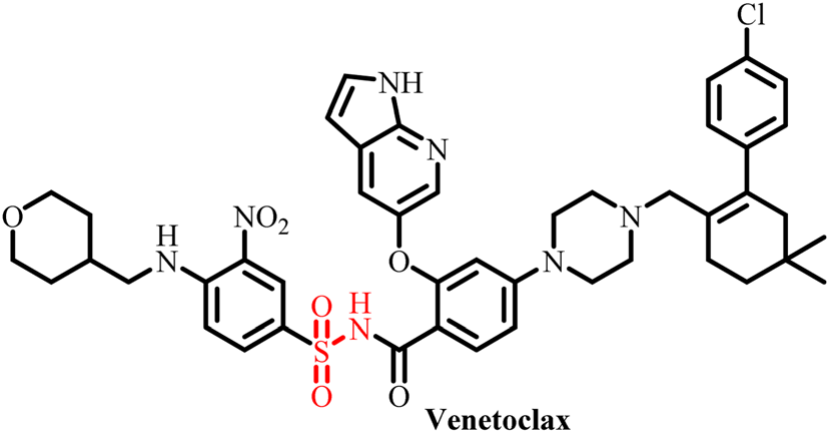	BCL-2 protein	Neutropenia, immune thrombocytopenia, febrile neutropenia, pneumonia, and upper respiratory tract infection	20–3300 nM	54

Sulfonamide derivatives have emerged as promising tubulin polymerization inhibitors for cancer therapy, owing to their structural versatility and strong binding affinity to the tubulin colchicine-binding site (Table 4).^55^ Their synthetic flexibility enables fine-tuning of molecular features to achieve optimal biological activity. The key attributes that make sulfonamide scaffolds attractive as potential MTAs include their ability to mimic the essential pharmacophoric elements of colchicine-site inhibitors,^56^ such as aromatic rings and hydrogen bond donor/acceptor functionalities, while offering improved aqueous solubility compared to classical hydrophobic tubulin inhibitors. Furthermore, rational structural modifications allow the enhancement of their selectivity, potency, and pharmacokinetic properties, making sulfonamide derivatives valuable candidates in anticancer drug design.

**Table 4:** Some tubulin-targeting sulfonamide-based drug candidates in clinical trials

S. no.	Drug	Clinical status	Used for	Clinical trial ID
1	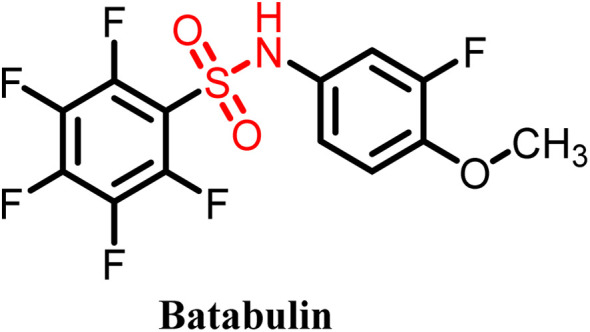	Phase II	Lung cancer	NCT00003359
2	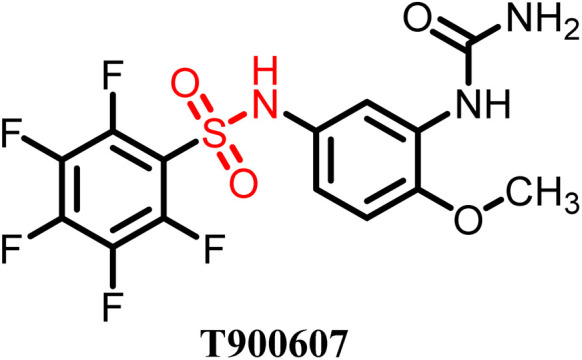	Phase II	Gastric and liver cancer	NCT00054249
3	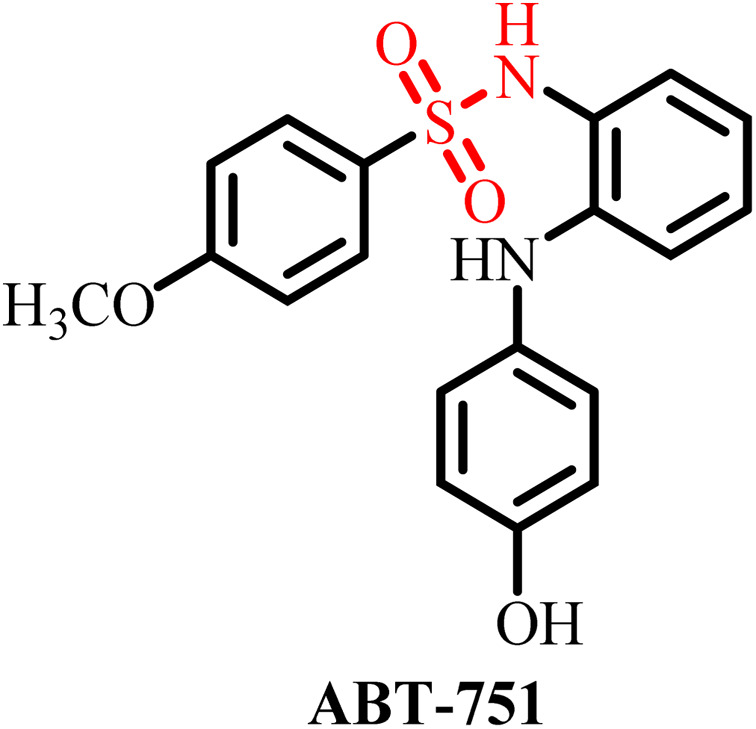	Phase II	Colorectal cancer	NCT00073138
4	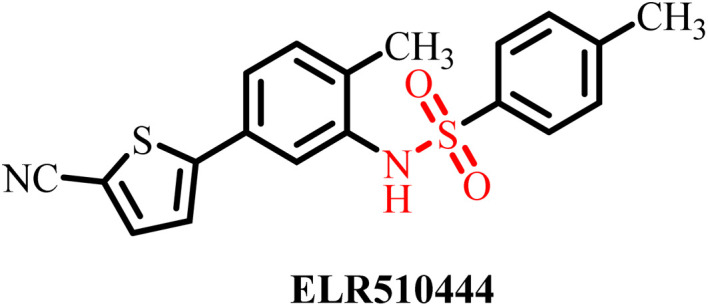	Preclinical	Renal cancer	57

A variety of sulfonamide derivatives are found to be active against several cancer cell lines including multidrug resistant cell lines. Thus, sulfonamide derivatives have potential to overcome or address the issue of further emerging resistance such as overexpression of efflux P-glycoprotein. Additionally, they provide an approach to refine pharmacokinetics and pharmacodynamic attributes by using the rational design strategy. The E7010 sulfonamide derivative showed broad-spectrum anticancer activity in a xenograft model and entered clinical trials. It is an orally bioavailable sulfonamide that binds to the colchicine site and investigated for its anticancer activity against several pediatric and adult cancers, showing modest efficacy with better toxicity profiles compared to vinca alkaloids. However, several significant drawbacks have been reported, including induction of stable disease rather than marked tumor regression and a short plasma half-life.^58^ E7070 is also a sulfonamide derivative that advanced to phase I and phase II clinical trials for various solid tumors but these trials were suspended in the mid-2000s due to its minor antitumor activity when used as a single agent.^59^ Although sulfonamide derivatives generally exhibit reduced toxicity compared to classical tubulin inhibitors, certain analogues still display cytotoxicity and suboptimal selectivity. Thus, despite the significant clinical data, no sulfonamide tubulin inhibitor has achieved FDA approval to date. To overcome these limitations, researchers have adopted multiple design strategies, including heteroaryl substitution to enhance colchicine-binding affinity, hybrid molecule development to achieve dual-targeting effects, and structure–activity relationship (SAR)-guided optimization to improve potency and pharmacokinetic behaviour.^49^ Additionally, advances in drug delivery, such as nanoparticle- or liposome-based encapsulation, are being integrated with hybrid drug design approaches to enhance bioavailability, tumor selectivity and toxicity reduction.^22^ These collective efforts are driving the evolution of next-generation sulfonamide tubulin inhibitors with improved therapeutic profiles and greater clinical promise.

### 2.1. Potential affinity of sulfonamide derivatives to bind at the colchicine site

**Fig. 3:**
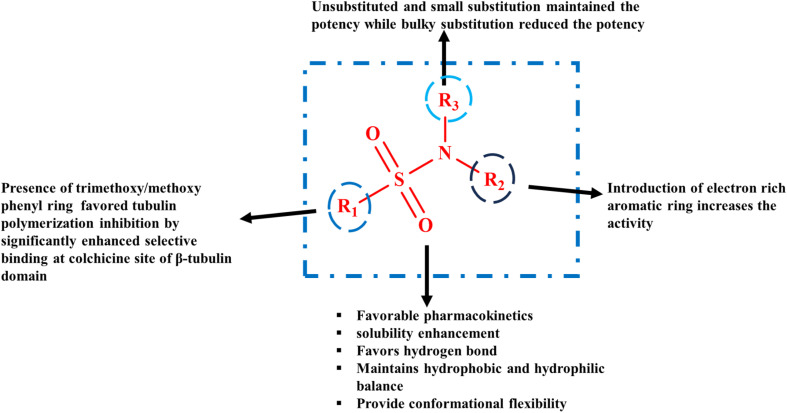
General SAR of sulfonamide derivatives for avoiding resistance and potential efficacy.

The presence of hydrophobic residues, which serve as major interaction points, makes the cavity of the colchicine site dominantly hydrophobic in nature.^60^ The ability of sulfonamide derivatives to form hydrogen bonds and hydrophobic interactions at the colchicine site of microtubules favours their binding at this site (Fig. 3). The sulfonamide group is an efficient bioisostere of the amide group with the ability to form hydrogen bonds. Thus, it is inserted as a linker by a number of researchers to form MTAs, which bind at the colchicine site to treat cancer.^61,62^ Combretastatin A-4 is a well-known prototype ligand for the colchicine site. However, the clinical translation of combretastatins has been limited due to several drawbacks. Diarylsulfonamide analogs have emerged as promising alternatives to CA-4 as antimitotic agents.^56^ Diarylsulfonamides have the potential to address the limitations of CA-4 as they can switch to the cisoid conformation to bind to the colchicine site, resulting in better pharmacokinetic profiles. The significant ability of sulfonamides to form strong hydrogen bonds with the amino acid residues of the colchicine site facilitates strong interactions between sulfonamide derivatives and the colchicine site. Sulfonamide derivatives also showed significant van der Waals interactions with the amino acid residues of the colchicine site, facilitating the accessibility of the sulfonamide group to the hydrophobic pocket of the colchicine site. Thus, the efficient chemical properties of sulfonamide derivatives allow the formation of favorable interactions of the developed sulfonamide derivative at CBS.

### 2.2. Toxicity and selectivity of sulfonamides compared with conventional MTAs^63^

The U. S. Food and Drug Administration approved the clinical use of paclitaxel (Taxol) in the late 1990s for the treatment of ovarian and metastatic breast cancers.^64^ However, despite the proven efficacy of taxanes, their clinical utility is often limited by their poor aqueous solubility, dose-limiting toxicities, and the development of drug resistance. To overcome solubility-related challenges, taxanes require complex solvent systems (*e.g.*, Cremophor EL-based formulations), which can alter their pharmacokinetic profiles and contribute to hypersensitivity reactions and systemic toxicity.

Mechanistically, taxanes exert their anticancer activity by stabilizing microtubule polymerization through binding to β-tubulin within microtubule protofilaments *via* lateral contacts, thereby preventing depolymerization at higher concentrations.^65^ At lower concentrations, taxanes suppress the microtubule dynamics, leading to mitotic arrest at the G2/M phase and subsequent apoptosis. Several additional drug-binding sites exist on tubulin that similarly modulate the microtubule dynamics, including the colchicine, vinca alkaloid, maytansine, and pironetin binding sites. However, among them, only agents targeting the vinca alkaloid site have achieved broad regulatory approval for cancer therapy, and they share limitations comparable to those of taxanes, such as neurotoxicity and resistance.

The colchicine-binding site on tubulin offers distinct advantages for the development of novel microtubule-targeting agents (MTAs). In addition to inhibiting microtubule polymerization, colchicine-site ligands often function as vascular-disrupting agents (VDAs), enhancing the selectivity and therapeutic efficacy against solid tumors by targeting tumor-associated vasculature. Notable colchicine-site inhibitors such as combretastatin A-4 (CA-4) and ABT-751 have advanced to clinical trials. Nevertheless, CA-4 suffers from chemical instability (*cis*–*trans* isomerization of the olefin bridge) and poor solubility, which limit its clinical application.

To address these shortcomings, González *et al.* (2021)^66^ investigated the replacement of the labile olefinic bridge in CA-4 with a sulfonamide linker and evaluated its impact on biological activity. The resulting sulfonamide-based MTAs demonstrated improved aqueous solubility and enhanced antiproliferative activity through effective inhibition of tubulin polymerization. The favorable pharmacokinetic and pharmacodynamic properties observed for these derivatives highlight the strategic value of introducing a sulfonamide bridge as a bioisosteric modification. Sulfonamide moieties are well known for their metabolic stability, hydrogen-bonding capacity, and ability to improve drug-like characteristics. Moreover, sulfonamide-containing compounds often display improved safety profiles and enhanced selectivity toward cancer cells, making them promising scaffolds for the development of next-generation colchicine-site microtubule inhibitors for cancer therapy. Table 5 summarizes the reasons why sulfonamide-based compounds are often considered superior in terms of toxicity and selectivity.

**Table 5:** Comparison of the toxicity profiles between sulfonamide and conventional MTAs

Feature	Sulfonamide MTAs (*e.g.*, T138067 and ABT-751)	Traditional MTAs (taxanes/vincas)
Binding site	Primarily the colchicine site (interfacial)	Taxane site (interior) or vinca site
P-gp sensitivity	Often evade P-glycoprotein efflux pumps (overcome MDR)	High substrate affinity for P-gp (lead to resistance)
Neurotoxicity	Generally lower; reduced affinity for axonal microtubules	High; frequently cause peripheral neuropathy
Selectivity	High selectivity for β-tubulin isoforms (overexpressed in tumors)	Broad binding across multiple tubulin isoforms in healthy tissues
Solubility	Enhanced through the polar sulfonamide moiety (–SO_2_NH_2_)	Often hydrophobic; require toxic vehicles (*e.g.*, paclitaxel)
Toxicity profile	Reversible binding leads to manageable hematological effects	Often irreversible or high-affinity binding, causing systemic toxicity

### 2.3. Clinical progress of sulfonamide derivatives as tubulin inhibitors

Clinical studies on developed sulfonamide derivatives have historically been limited due to their significant toxicities. Major limitations include bone marrow suppression, which emerges as dose-limiting toxicity (DLT), and peripheral neuropathy, contributing substantially to a decline in quality of life (QOL) in cancer patients.^67^ These serious adverse effects have led to the suspension of several sulfonamide derivatives during clinical studies such as E7070 and ABT-751. To overcome these limitations, recent efforts and research have been focused on the rational design and development of a new generation of sulfonamide derivatives with improved tumor selectivity and reduced off-target toxicity. Structural modifications have been explored to enhance the binding specificity to tubulin isoforms preferentially expressed in tumor cells, to reduce their systemic toxicity.^68^

Recent advances in conjugation and delivery strategies, such as sulfonamide–antibody conjugates and prodrug approaches, are being actively explored to achieve targeted delivery and mitigate dose-limiting bone marrow toxicity. Preclinical evaluations of several next-generation sulfonamide derivatives have demonstrated reduced neurotoxicity and hematological adverse effects while retaining potent anticancer efficacy.^69^ Importantly, understanding the clinical toxicities associated with microtubule-targeting agents (MTAs) provides valuable insights for the rational design of safer sulfonamide-based tubulin binders. For instance, eribulin, a clinically approved MTA, acts by binding to the plus end of microtubules, inhibiting polymerization and promoting the formation of non-productive tubulin aggregates.^70^ This mechanism, while effective in tumor suppression, is associated with peripheral neuropathy arising primarily from disrupted axonal microtubule dynamics and impaired axonal transport rather than general cytotoxicity.^71^

These mechanistic studies highlight that, beyond overall tubulin-binding potency, the selectivity toward specific tubulin isoforms and the topology of the binding site play crucial roles in determining both therapeutic efficacy and neurotoxicity.^72^ Notably, β-tubulin isotypes (*e.g.*, βIII-tubulin) exhibit differential expression patterns between neuronal and tumor cells, and selective targeting of particular isotypes may help balance antitumor activity with reduced peripheral neuropathy risk.^73,74^ Consequently, current research focuses on modifying sulfonamide-based tubulin binders through preclinical profiling to enhance their tumor selectivity while minimizing neuronal damage in rodent neuropathy models.

A notable example is E7820, a benzene-sulfonamide derivative conjugated with a bioactive indole moiety, which recently entered phase II clinical evaluation.^75^ Although not a classical tubulin binder, E7820 functions as a molecular glue that induces the degradation of the RNA splicing factor RBM39, demonstrating an innovative mechanism of anticancer action. These findings underscore that strategic molecular redesign and conjugation-based delivery approaches can overcome the toxicological limitations of earlier sulfonamide-based MTAs, thereby supporting their continued clinical advancement.

The rising global incidence of cancer, along with limitations of current treatment procedures, underscores the need for novel chemotherapeutic agents that minimize toxicity toward normal cells. As a result of their ability to disrupt microtubule dynamics and mitotic progression, significant progress has been made in developing tubulin polymerization inhibitors as potential anticancer agents.^76^ In this review, we highlight recent advances in sulfonamide-based drug candidates, with particular emphasis on research from 2018–2025. Sulfonamide derivatives have gained attention due to their favourable physicochemical properties and ability to interact with functional tubulin sites. We discuss their mechanisms of action, structure–activity relationships (SAR), structural diversity, and other features contributing to their anticancer efficacy. Additionally, we outline promising next-generation sulfonamide analogues and drug conjugates reported in the recent literature. This focused account aims to provide valuable insights for the rational design and development of newer tubulin-targeting agents based on the sulfonamide scaffold.

## 3. Structure–activity relationship (SAR) analysis of sulfonamide-based tubulin inhibitors

Systematic modifications of sulfonamide scaffolds have revealed key structural features governing tubulin-binding affinity and antiproliferative activity.

### 3.1. Aromatic core

Aromatic rings with electron-rich substituents (4-methoxy, amino, and hydroxyl) show enhanced tubulin-binding affinity by providing strong interactions within the binding pocket.^77^ Smaller electron-donating substituents such as methyl, methoxy, and amine (hydrogen bond donor) increase the potency of drug candidates in comparison to bulky groups.^66^

### 3.2. Sulfonamide linkage

The –SO_2_–NH– linker is crucial for anchoring *via* hydrogen bonding with key residues (Cys241β and Ala250β). Substitution of the NH group or inversion of the sulfonyl oxygen orientation significantly decreases activity.^78^

### 3.3. Amide/carbonyl extensions

Incorporation of flexible amide or heteroatom linkers improves conformational adaptability, favouring the optimal orientation in the colchicine-binding pocket and enhanced activity.^79^

### 3.4. Heterocyclic substituents

Introduction of indole, quinazoline, pyrimidine, *etc.* fragments has been shown to improve lipophilicity and cell permeability, translating to stronger antiproliferative effects in resistant cell lines.^80^

### 3.5. Correlation with biological activity

IC_50_ values in human tumor cell lines correlate strongly with predicted docking scores and hydrogen-bond occupancy in the β-tubulin binding site. This supports the use of computational modeling as a predictive design tool for next-generation sulfonamides.

## 4. Design implications

These structure–activity relationship (SAR) insights highlight that preserving the core sulfonamide linkage while strategically modifying substituents on the aromatic and heterocyclic frameworks is essential to achieve an optimal balance among potency, selectivity, and pharmacokinetic stability. Rational drug design approaches that integrate *in silico* modeling, SAR-guided optimization, and comprehensive biological evaluation can significantly enhance the development of more effective and economically viable sulfonamide-based drug candidates. In light of these findings, several recent studies have reported new sulfonamide derivatives with improved anticancer efficacy and reduced toxicity profiles, as summarized below.

**Fig. 4:**
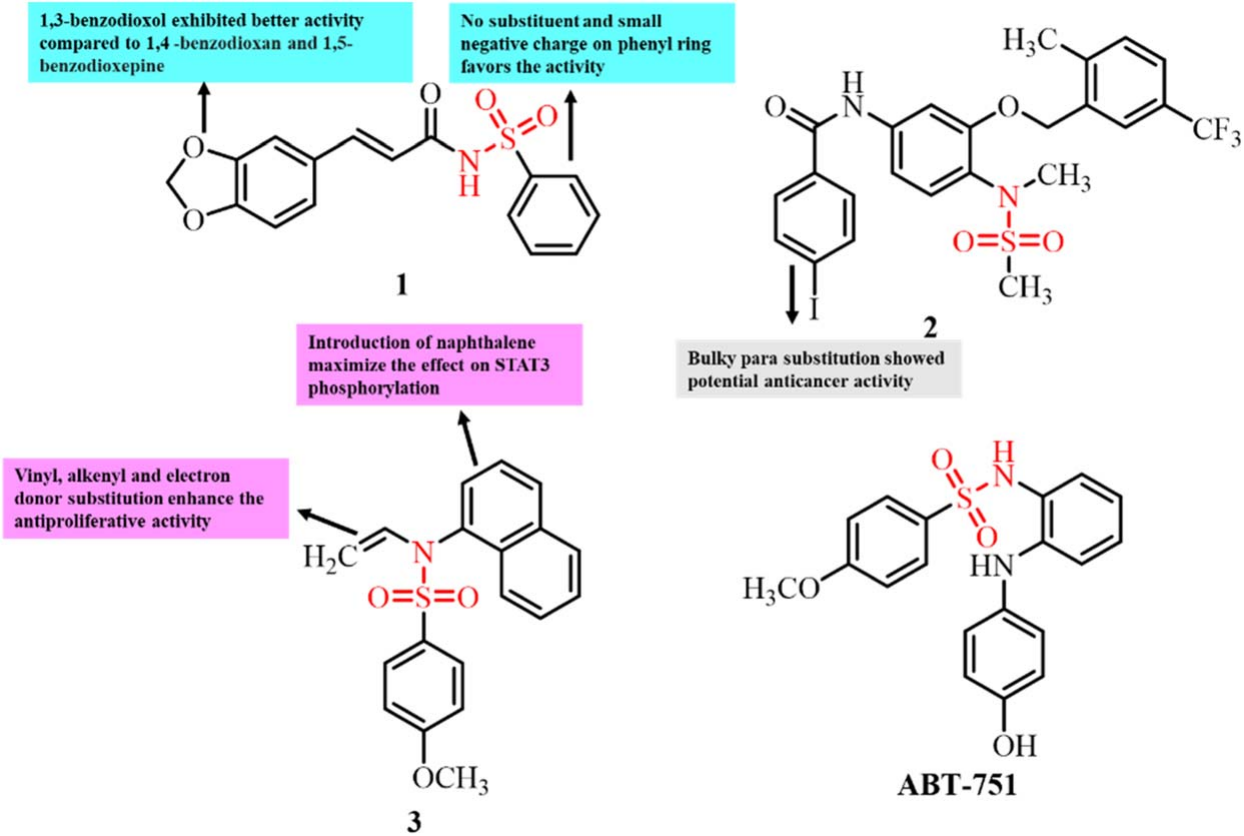
Chemical structures of sulfonamide derivates 1–3 and ABT-751.

Luo *et al.* designed and developed a novel series of cinnamic acyl sulfonamide derivatives and investigated their anti-proliferative activity and anti-tubulin polymerization potential.^81^ Compound 1 displayed most significant results as a tubulin inhibitor having an IC_50_ value of 0.88 µM. Moreover, compound 1 exhibited the most significant results for antitumor activity against the MCF-7 cancer cell line with an IC_50_ value of 0.17 µg mL^−1^ (Fig. 4). Molecular docking studies of compound 1 at the colchicine site of tubulin revealed that interactions of this compound are favored by hydrogen bonds and van der Waals forces. These interactions allowed the accessibility of the developed molecule selectively to the hydrophobic pocket of tubulin. Thus, compound 1 has potential for further development as a lead for the inhibition of tubulin protein.

Jaragh-Alhadad *et al.* designed and developed sulfonamide derivatives and investigated their anticancer and tubulin polymerization inhibition potential.^80^ The developed sulfonamide derivatives displayed significant anticancer activity against both MCF7 and A549 cancer cell lines. Among them, compound 2 having an iodo benzoyl substituent displayed the most promising results, having IC_50_ of values less than 1.8 µM against the tested cancer cell lines (Fig. 4). Both the MCF7 and A549 cancer cell lines showed overexpressed tubulin levels. To confirm the action of these compounds through inhibiting tubulin polymerization, a western blot assay was performed. The results showed disruption of the microtubule network on treatment with compound 2, which confirmed that the tested compound is a potent tubulin binding agent and causes inhibition of tubulin polymerization. Thus, the developed sulfonamide derivatives exhibited anticancer activity by targeting tubulin polymerization. Hence, sulfonamide conjugate 2 is a potential tubulin inhibitor and can serve as a lead for the development of future tubulin-targeting anticancer therapeutics.

Wang *et al.* developed a series of 4-methoxy-*N*-(1-naphthyl)benzenesulfonamide derivatives as potential dual-target inhibitors of tubulin and STAT3.^82^ Among them, compound 3 exhibited significant anticancer activity against β-tubulin and STAT3 in A549, HCT-116, and MDA-MB-231 cell lines, with IC_50_ values of 1.35, 2.85, and 3.04 µM, respectively (Fig. 3). It also showed comparable tubulin polymerization inhibition and colchicine-binding site affinity to ABT-751 (Fig. 4), with dose-dependent inhibition activity and an IC_50_ of 0.83 µM. Furthermore, *in vivo* studies revealed that 3 suppressed tumor xenograft growth by over 80%, which is attributed to its STAT3 inhibitory action. These findings highlight the promising results of sulfonamide-based compounds as effective dual-target anticancer agents.

**Fig. 5:**
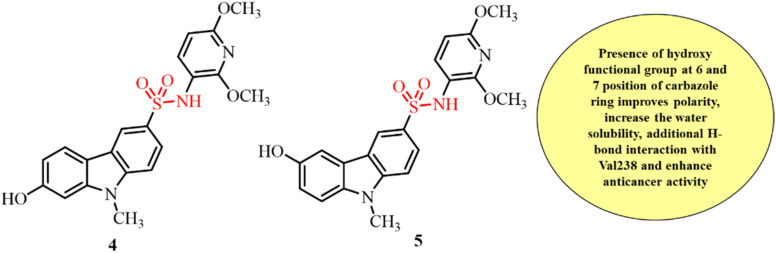
Chemical structures of carbazole–sulfonamide conjugates 4 and 5.

Esophageal squamous cell carcinoma (ESCC) is one of the most common and leading causes of cancer mortality worldwide. Its chemoresistance and poor prognosis make it even more severe. Targeting microtubule polymerization is an effective approach for the treatment of this cancer. In 2018, Niu and coworkers investigated the *in vitro* and *in vivo* anticancer activity of two small carbazole sulfonamide derivatives against ESCC and their ability to inhibit tubulin polymerization (Fig. 5).^83^ Mechanistic studies revealed that both compounds 4 and 5 exerted significant anticancer activity by inducing cell apoptosis and cell cycle arrest in the G2/M phase in both dose- and time-dependent manners. Western blot assays revealed that both compounds potentially inhibit microtubule polymerization by down regulating acetylated α-tubulin. Consistent with the *in vitro* findings, both compounds significantly inhibited tumor growth and reduced microvessel density in an ESCC xenograft model *in vivo*. Both compounds showed no stimulation of Pgp ATPase activity and thus have the potential to overcome the issue of drug resistance. Hence, these studies revealed that these compounds have potential anticancer activity and may even be better than many preclinical drugs for development as novel chemotherapeutic and antimitotic agents for the treatment of ESCC in the future.

**Fig. 6:**
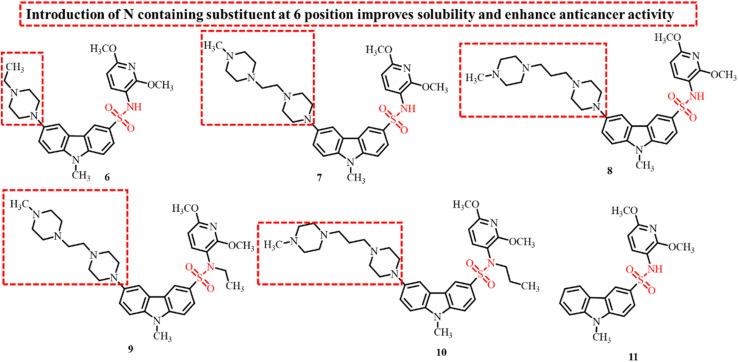
Chemical structures of carbazole sulfonamide derivatives with different substituents at the 6-position of carbazole ring as anticancer agents.

Sun *et al.*, in 2020, constructed a series of novel carbazole sulfonamides with varying substituents on the carbazole ring as potential anticancer and antitubulin agents.^84^ These derivatives were screened for their anticancer activity against HepG2, MCF-7, MIA PaCa-2, and Bel-7402 cell lines. These compounds showed 2-5-times more potent activity against the HepG2 cell line in comparison to the other three tested cell lines with IC_50_ values in the range of 1–10 µM. The representative compounds 6, 7, 8, 9 and 10 were further evaluated to investigate their potential to inhibit tubulin polymerization in comparison to the reference compound 11 (Fig. 6). The results revealed the poor affinity of the tested compounds towards tubulin inhibition at 10 µM concentration. The inhibition efficiency of the compounds slightly improved at a concentration of 100 µM, reaching approximately 50% for most compounds, except for 8, which exhibited a significantly higher inhibition rate of 84%. These results suggest that, although the developed compounds are derivatives of known tubulin inhibitors, their structural modifications led to a reduction in their ability to inhibit microtubule polymerization.

**Fig. 7:**
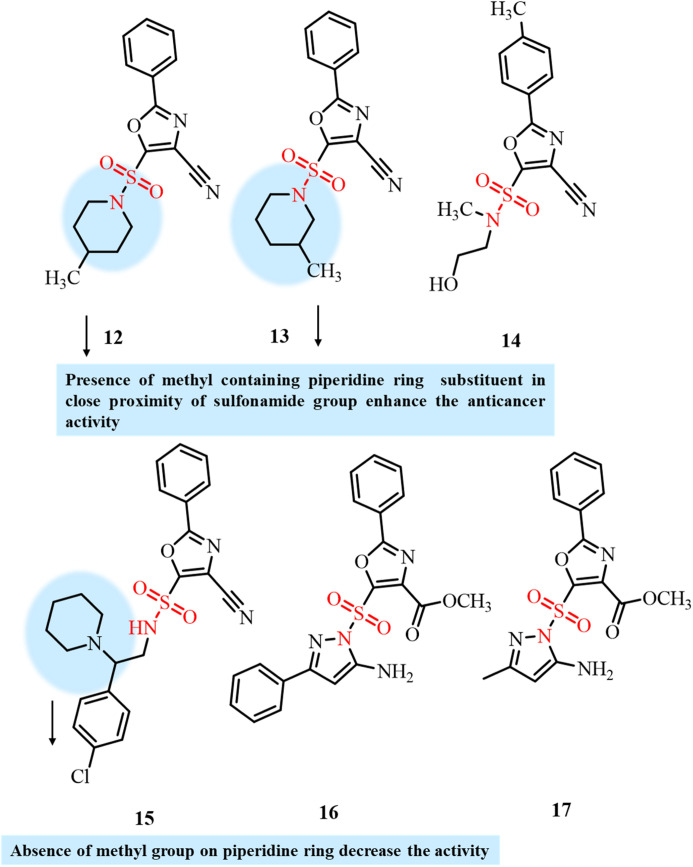
Chemical structures of antimitotic oxazole sulfonamide derivatives 12–17.

Based on the excellent anticancer activity of the known anticancer agent E7010, Kachaeva *et al.* reported the rational design of some novel derivatives of the E7010 scaffold using the QSAR modeling method and carried out their synthesis.^85^ QSAR studies revealed the potential tubulin polymerization inhibition activity of the newly designed sulfonamides as antiproliferative agents. The developed derivatives were screened for anticancer activity against 60 different human cancer cell lines. Compounds 12–17 displayed potent anticancer activity against several tested cancer cell lines (Fig. 7). In comparison to the cytotoxic activity of 12 and 13, compound 15 displayed relatively lower anticancer activity and mainly cytostatic activity, which might be due to the presence of a piperidine ring farther from the sulfonamide group, while that in the case of 12 and 13 is closer to the sulfonamide group. Docking studies revealed that these derivatives exhibited significant activity by inhibiting tubulin polymerization through their significant interactions at the colchicine site. Hence, compounds 12–17 have the potential to become anticancer agents by targeting tubulin polymerization.

**Fig. 8:**
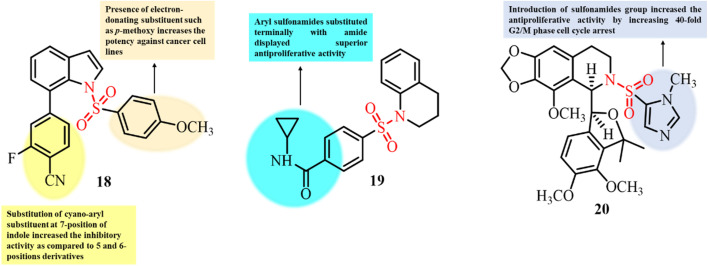
Chemical structures and SAR of sulfonamide derivatives 18–20.

Qiao and colleagues reported the synthesis of *N*-arylsulfonyl-substituted 1*H*-indole derivatives and investigated their antiproliferative activity against a range of cancer cell lines. Several compounds demonstrated potent anticancer activity, among which compound 18 showed the most promising results, exhibiting IC_50_ values below 10 µM across all the tested cell lines.^86^ Immunofluorescence staining and tubulin polymerization inhibition assays indicated that compound 18 caused cell cycle arrest at the G2/M phase by inhibition of tubulin polymerization, leading to apoptosis (Fig. 8). At a concentration of 5 µM, 18 significantly disrupted β-tubulin assembly in a dose-dependent manner, similar to the mechanism of colchicine. These findings suggest that compound 18 exerts its antiproliferative effects primarily through the inhibition of tubulin polymerization.

Field *et al.* reported the synthesis of seven novel small-molecule aryl sulfonamides substituted with amide and carboxylic acid groups. The developed derivatives were investigated for their anticancer activity against two human cancer cell lines, *viz.* HL-60 and 1A9.^87^ The anticancer activity results showed that substitution with an amide at the *para* position, in comparison to a carboxylic acid group, favors antiproliferative activity. The most potent compound 19 was further analyzed against three other cancer cell lines. The significant IC_50_ values of compound 19 in the low micromolar range indicate its potential as an antiproliferative agent (Fig. 8). The effect of 19 on cell cycle arrest was further analyzed by flow cytometry. The results showed significant blockage of 1A9 cells in the G2/M phase of the cell cycle, indicating potential antimitotic activity. An intracellular tubulin inhibition assay indicated that 19 caused significant inhibition of tubulin polymerization in the micromolar range. The anti-tubulin activity of compound 19 was further confirmed by an *in vitro* tubulin polymerization assay, which demonstrated its direct interaction with brain tubulin and significant inhibition of tubulin polymerization, even in the presence of glycerol. These findings suggest that compound 19 has potential antimitotic activity and significant anticancer activity.

Over the years, extensive structural modifications have been made to noscapine, a natural compound with weak anticancer efficacy, to develop more potent analogues. Yong *et al.* further advanced these efforts by synthesizing noscapine derivatives bearing *N*-sulfonyl and *N*-sulfamoyl substituents.^88^ These analogues demonstrated enhanced anticancer efficacy and improved mitotic arrest compared to the parent compound. Notably, compound 20, featuring an *N*-methylimidazol-4-yl substituent, displayed the most potent antiproliferative activity, with EC_50_ values in the nanomolar range across several human cancer cell lines (Fig. 8). *In vitro* studies revealed that 20 reduced tubulin polymerization by eight-fold. Interaction assays showed that paclitaxel increased polymerization threefold, while noscapine reduced the polymerization rate (*V*_max_ = 18.05 ± 0.98 mOD per min). In contrast, 20 (10 µM) decreased the rate significantly to 3.241 ± 0.048 mOD per min and remained active throughout the experiment, unlike the other ligands, which reached a steady state by 20 min. Importantly, 20 retained its antiproliferative activity in drug-resistant cancer cells overexpressing P-glycoprotein. These findings highlight 20 as the most effective noscapine derivative reported to date, with strong potential as an antimitotic and anticancer agent.

**Fig. 9:**
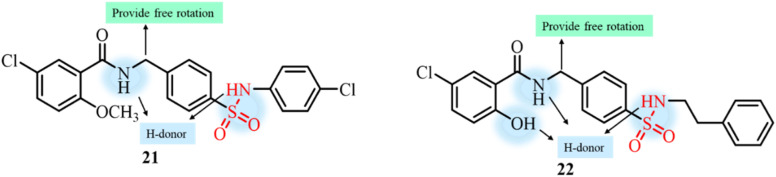
Chemical structures of salicylamide-linked sulfonamide derivatives 21 and 22.

Galal *et al.*, in 2018, reported the development of a novel series of sulfonamide derivatives linked with a salicylamide scaffold (Fig. 9).^89^ The developed compounds were investigated for antiproliferative activity against five different human cancer cell lines, where nine of the tailored derivatives displayed potent growth inhibition of four of the tested cancer cell lines. Compound 22 displayed significant activity against colon cancer cell lines, while 21 showed the most potent activity against breast cancer. *In vitro* polymerization and flow cytometric assays revealed that the compounds caused cell cycle arrest in the G2/M phase potentially by hindering the tubulin polymerization process in a dose-dependent manner. Docking experiments also revealed binding of the compounds in the colchicine site of the tubulin protein. The binding interactions of the most potent compounds in the inhibitory pocket of tubulin ranged from −10.2 to −10.9 kcal mol^−1^, and these values are better than that of the known standard tubulin inhibitor colchicine, which has a binding affinity of −9.5 kcal mol^−1^. The formation of strong hydrogen bonds between the hydroxy groups of the potent compounds and surrounding amino acids is responsible for the increased affinity of the developed compounds for binding with the inhibitory pocket compared with that of colchicine.

**Fig. 10:**
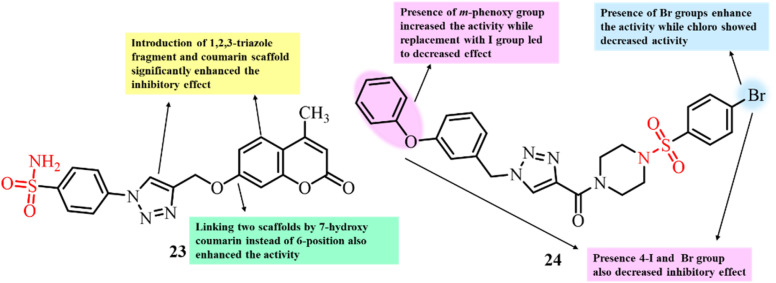
Chemical structures and SAR of triazole-linked sulfonamide derivatives 23 and 24.

Guo *et al.* reported the development of sulfanilamide–1,2,3-triazole hybrids as novel anticancer agents (Fig. 10). The antiproliferative activity of these hybrid compounds was evaluated against three cancer cell lines: BGC-823, MGC-803, and SGC-790.^90^ All the tested derivatives exhibited significant anticancer potential, with compound 23, bearing both coumarin and triazole moieties, showing the most significant activity, particularly for the MGC-803 cell line (IC_50_ = 0.4 µM). *In vivo* studies further exhibited that 23 significantly reduced tumour growth in a xenograft model. Additionally, *in vitro* tubulin polymerization assays displayed that 23 inhibited tubulin polymerization by 50%, with an IC_50_ value of 2.4 µM. These findings highlight sulfanilamide–1,2,3-triazole hybrids, particularly compound 23, as promising antimitotic agents with strong anticancer potential.

Manasa *et al.* (2020) designed and synthesized a novel series of substituted (1-(benzyl)-1*H*-1,2,3-triazol-4-yl)(piperazin-1-yl)methanone derivatives with a sulfonamide group and evaluated their anticancer activity (Fig. 10).^91^ Among them, compound 24, featuring *m*-phenoxy and bromo substituents, showed the highest cytotoxicity against the BT-474 cancer cell line (IC_50_ = 0.99 ± 0.01 µM). Clonogenic assays confirmed concentration-dependent inhibition of colony formation, while flow cytometry revealed cell cycle arrest at the sub-G1 and G2/M phases. Tubulin polymerization assays and molecular docking studies indicated that 24 binds at the colchicine-binding site of tubulin. Key interactions included hydrogen bonding between the sulfonyl and triazole groups with Ser178, Ala250, and Lys254, along with stabilizing hydrophobic interactions. These findings demonstrate that 24 acts as a potent antimitotic agent and provide valuable insights for the rational design of future tubulin polymerization inhibitors.

**Fig. 11:**
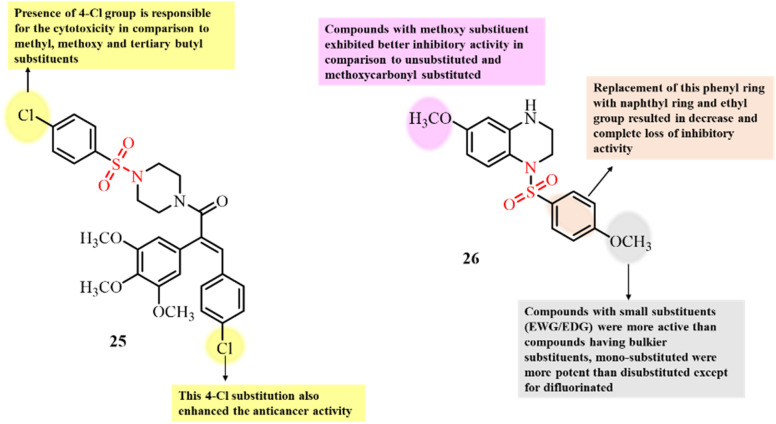
Chemical structures of combretastatin A4 acid sulfonamide derivative 25 and tetrahydroquinoxalin sulfonamide 26.

To incorporate the biological activity of two bioactive heterocyclic scaffolds into one structure, the molecular hybridization approach has proven to be the most significant strategy for enhancing biological activity. Employing this approach, Jadala *et al.* tailored a molecular hybrid having combretastatin A4 acid and sulfonyl piperazine scaffolds (Fig. 11). The developed compounds were screened for their anticancer activity against several cancer cell lines.^92^ Among the tested cell lines, compound 25 displayed the most potent activity, with IC_50_ values in the range of 0.36 to 7.08 µM against the tested cancer cell lines. Further mechanistic investigations revealed that 25 inhibited the tubulin polymerization process (IC_50_ = 5.24 ± 0.02 µM). Compound 25 significantly inhibited 50% of tubulin polymerization at 10 µM concentration. Molecular docking studies revealed the significant binding affinity of 25 to the colchicine site, which is responsible for its inhibition of tubulin polymerization. Hydrogen bonds and hydrophobic interactions between amino acid residues and ligand 25 were responsible for its significant binding. Results of a flow cytometric assay revealed that 25 induced cell cycle arrest at the G2/M phase in A549 cells. Thus, a novel series of compounds with significant microtubule inhibition activity has been developed for the treatment of cancer.

The development of colchicine binding site inhibitors is a significant strategy as they inhibit microtubule polymerization and thus have the potential to overcome drug resistance, ultimately enhancing the efficacy of cancer treatment. Dong *et al.*, in 2023, designed and developed novel tetrahydroquinoxalin sulfonamide derivatives and investigated their antiproliferative activity against four cancer cell lines (Fig. 11).^62^ The results showed that some of the tested compounds displayed moderate to strong anticancer activity against different cancer cell lines including HT-29, HePG2, HeLa and MCF-7. Compound 26 was the most potent among the tested compounds, with IC_50_ values in the range of 2.20–7.52 µM for the tested cancer cell lines. Moreover, 26 caused significant inhibition of tubulin polymerization, disruption of the microtubule network and mitotic spindle cell cycle arrest in the G2/M phase of the cell cycle. Molecular docking studies revealed that compound 26 binds at the colchicine-binding site in the inhibitory pocket of tubulin through strong hydrogen bonds and hydrophobic interactions.

**Fig. 12:**
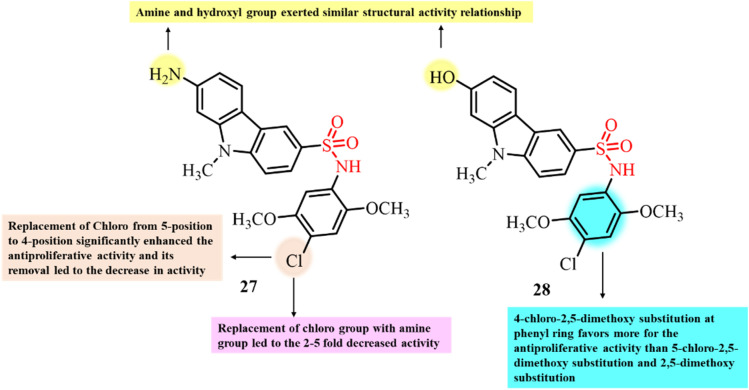
Chemical structures of carbazole sulfonamide derivatives 27 and 28.

Liu *et al.*, in 2024, designed and developed a novel series of carbazole sulfonamide derivatives and investigated their anticancer activity (Fig. 12).^93^ Compounds 27 and 28, bearing 4-chloro-2,5-dimethoxyphenyl substituents, showed the most significant activity, with IC_50_ values in the range of 0.81–31.9 nM against five tested human cancer cell lines, namely Bel-7402, MDA-MB-231, MIA PaCa-2, NCI-H460 and KB. Compound 28 showed excellent selectivity for cancer cells, with a selectivity index of 7.7. The valine and phosphate prodrugs of 27 and 28, respectively, also displayed significant inhibition of tumor growth. SAR studies revealed that the 4-chloro-2,5-dimethoxy substituent on the phenyl ring and 7-OH group on the carbazole ring played crucial roles in the antiproliferative activity. Further mechanistic studies revealed the direct binding of both the molecules to the colchicine site *via* hydrogen bonding interactions, inhibiting the tubulin polymerization process (IC_50_ = 5 nM) and ultimately causing cell death through cell cycle arrest in the G2/M phase. Computational studies revealed that 27 and 28 directly bind to tubulin at the colchicine site. Thus, 27 and 28 have the potential to serve as tubulin inhibitors for cancer therapeutics.

**Fig. 13:**
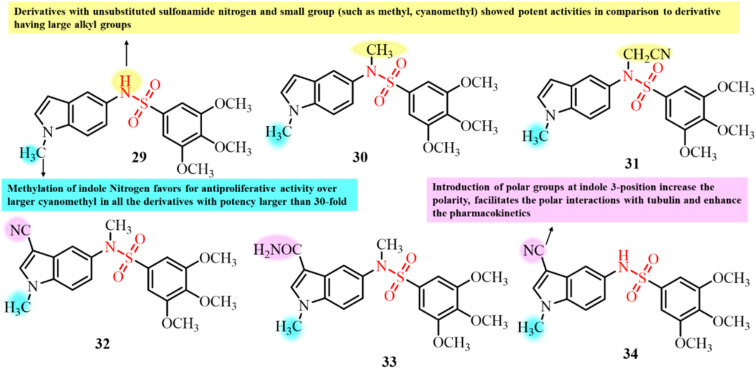
Chemical structures of *N*-indolyl-3,4,5-trimethoxybenzenesulfonamide derivatives 29–34.

Fuentes-Martín *et al.* (2024) developed a series of *N*-indolyl-3,4,5-trimethoxybenzenesulfonamide derivatives by substituting the sulfonamide nitrogen and the indole ring at positions 1 and 3 (Fig. 13). Initial screening against HeLa cells identified compounds with nanomolar IC_50_ values, which were further evaluated across a broader panel of cancer cell lines.^56^ SAR analysis revealed that methyl substitution enhanced antiproliferative activity, while modifications at the indole-3 position showed no benefit. Unsubstituted or short-chain sulfonamide substituents favoured antiproliferative activity, whereas bulky groups reduced the efficacy. The most potent compounds (29, 30, 31, 32, 33, and 34) induced G2/M phase cell cycle arrest and caspase 3/7 activation. The compounds with sulfonamide substituents promoted apoptosis *via* mitotic arrest, while unsubstituted and cyanomethyl derivatives led to necrotic cell death. Immunofluorescence assays at 10 nM demonstrated microtubule depolymerization and loss of cellular structure. Notably, compounds 30, 32, and 33 exhibited tubulin inhibition activity comparable to vincristine, highlighting their potential as antimitotic agents.

**Fig. 14:**
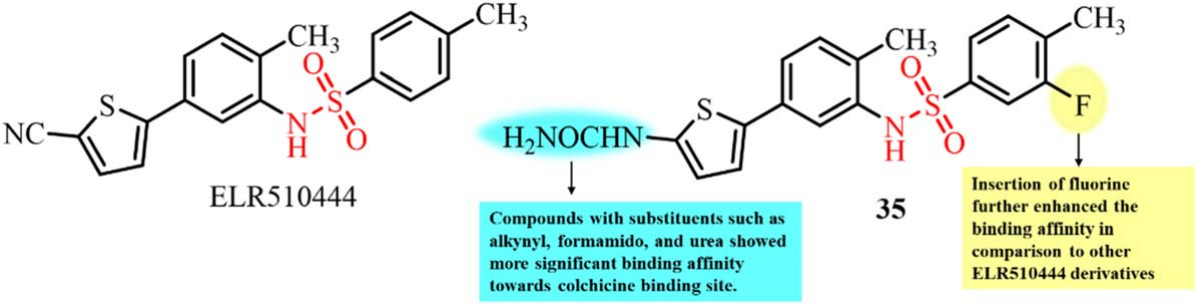
Chemical structures of the known tubulin inhibitor ELR510444 and its derivative 35.

To design and develop more effective therapeutic candidates for treating cancer by targeting microtubules, Lei *et al.*, in 2021, published a study providing structural insights into the crystal structure of tubulin complexed with the known highly potent colchicine site binder and tubulin inhibitor ELR510444 (Fig. 14).^94^ Molecular docking studies revealed that ELR510444 binds at the colchicine site more efficiently than colchicine *via* hydrophobic interactions with the Val181 residue of α-tubulin. The cyano-group of ELR510444 significantly extended the interaction with Asn165 for efficient activity with a binding affinity score of −11.1 kcal mol^−1^. SAR through molecular docking studies showed that compound 35 with the predicted best substituents displayed superior binding affinity compared to other ELR510444 derivatives (Fig. 13). Thus, this research article provides a strong foundation to develop anti-tubulin agents that significantly bind to the colchicine site and efficiently inhibit tubulin polymerization for cancer therapy.

**Fig. 15:**
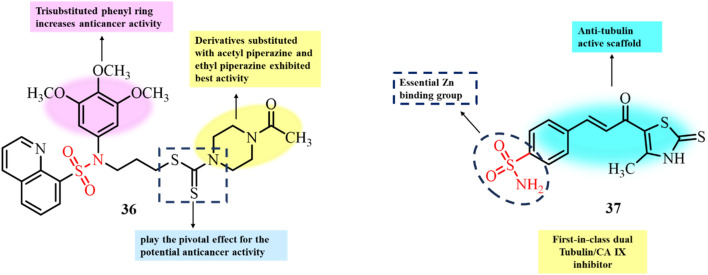
Chemical structures of sulfonamide–dithiocarbamate (36) and thiazole–sulfonamide (37) hybrids.

Bladder cancer is one of the most expensive malignancies to treat due to the need for lifelong clinical management. Bladder cancer is difficult to treat primarily due to its high metastasis rate. This necessitates the urgent development of novel anticancer agents for the treatment of bladder cancer. In 2019, Liu *et al.* reported the development of novel sulfonamide–dithiocarbamate hybrids using a conventional molecular hybridization approach (Fig. 15). Among the synthesized derivatives, compound 36, bearing an acetyl substituent on the piperazine ring, exhibited the most potent anticancer activity, with IC_50_ values of 0.9, 0.7, 1.9, and 2.6 µM against the UM-UC-3, RT-112, RT4, and T24 bladder cancer cell lines, respectively.^95^ Compound 36 also significantly inhibited cell proliferation in a concentration- and time-dependent manner. *In vitro* microtubule polymerization and immunofluorescence staining assays revealed that 36 effectively disrupted microtubule structures in RT-112 cells, with an IC_50_ of 2.9 µM for tubulin inhibition. Furthermore, treatment with 36 significantly reduced tumor growth in an RT-112 xenograft model, with minimal toxicity. These findings suggest that 36 is a promising lead compound for bladder cancer therapy *via* microtubule inhibition.

Recently, Khasawneh *et al.* reported the synthesis and biological evaluation of novel thiazole–sulfonamide conjugate molecules (Fig. 15).^63^ The developed conjugates were investigated for their antiproliferative activity, among which compound 37 was found to be the most potent and selective only for the HT-29 cancer cell line with an IC_50_ value of 0.98 µM. Tubulin polymerization assay results for compound 37 depicted its significant tubulin inhibition, with an IC_50_ value of 2.72 µM. Compound 37 also showed potential inhibition activity against CA IX (IC_50_ = 0.021 µM). The lower IC_50_ value for the antiproliferative activity of the tested compound compared to the IC_50_ for its tubulin inhibitory activity is due to its dual-targeting mechanism for anticancer activity. They also reported that, in comparison to their previous finding, structural optimization and substitution of the sulfonamide group at *para* position enhanced the tubulin inhibitory potency. The incorporation of electron-withdrawing groups further enhanced the binding affinity of 37 with the colchicine-binding site of tubulin by providing additional hydrogen bonding and hydrophobic interactions.

**Fig. 16:**
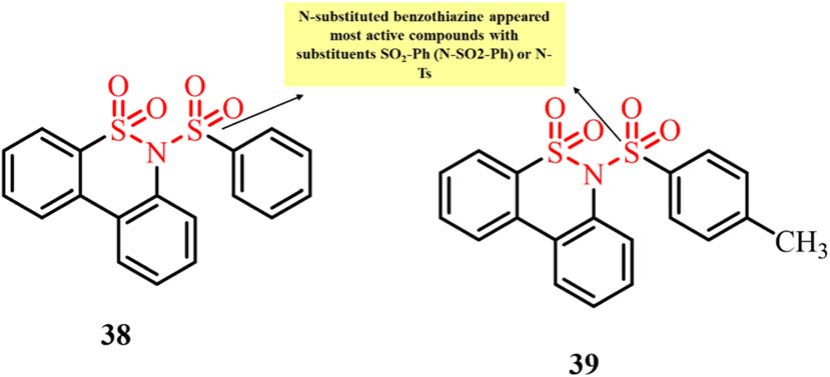
Chemical structures of dibenzothiazine-incorporated sulfonamide derivatives 38 and 39.

In 2021, Guerra *et al.* reported the synthesis of novel heterocyclic scaffolds based on dibenzothiazines incorporated with a sulfonamide functional group (Fig. 16). The developed derivatives were screened for their anticancer activity against six human cancer cell lines, as well as their ability to inhibit tubulin polymerization. Among them, *N*-substituted derivatives 38 and 39 showed the most potent activity, with GI_50_ values ranging from 2 to 5.4 µM.^96^ Cell cycle analysis indicated arrest at the G2/M phase, consistent with tubulin polymerization inhibition. Moreover, *in vitro* tubulin polymerization assays confirmed that both compounds effectively disrupted tubulin assembly. Further studies suggested that these compounds exert their anti-tubulin activity through a blocking mechanism by binding to the colchicine site, rather than acting through a tubulin-poisoning mechanism. These discoveries highlight the significance of dibenzothiazine–sulphonamide hybrids as promising antimitotic agents for cancer therapy.

**Fig. 17:**
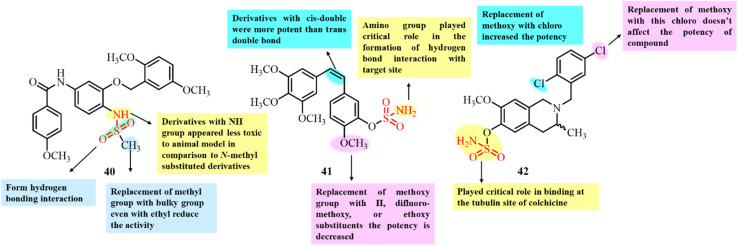
Chemical structures of developed sulfonamide derivatives 40–42.

Li *et al.*, in 2021, reported the potential of compound 40 to target both tubulin and HSP27 for the treatment of glioblastoma (Fig. 17). Initially, they studied the binding of compound 40 to Hsap27 to induce androgen receptor degradation for the treatment of glioblastoma.^97^ However, they also observed inhibition of cell proliferation of glioblastoma cells without AR expression, indicating the general activity of this compound against GBM cells. Thus, they further examined the tubulin polymerization inhibition potential of compound 40. An *in vitro* tubulin polymerization assay showed a dose-dependent inhibition of tubulin polymerization by compound 40 at a concentration of 1 µM, which was even more significant than that of the positive control nocodazole, suggesting that compound 40 is a potent tubulin inhibitor for cancer treatment. Docking studies also revealed the significant binding of compound 40 to the colchicine-binding site of tubulin. The docking results revealed significant hydrogen bonding interactions between the sulfonamide group of 40 and the Ala316 and Lys352 residues of tubulin. Thus, the significant binding interaction favors the potential inhibition by 40 for future chemotherapeutic agent.

Huang *et al.* (2020) reported the synthesis of combretastatin A-4 (CA-4) sulfamate derivatives and evaluated their antiproliferative activity (Fig. 17). Among the synthesized compounds, 41 exhibited the most potent growth inhibition across the tested cancer cell lines.^98^ Molecular docking studies further revealed that the sulfamate group of compound 41 formed strong hydrogen bond interactions with the colchicine-binding site of tubulin, specifically amino acid residues Aala180 and Asn349. These interactions suggest that 41 functions as a tubulin polymerization inhibitor. Thus, compound 41 shows significant potential as an anticancer agent and may serve as a promising lead for the development of novel tubulin-targeting anticancer drugs.

Dohle *et al.*, in 2019, reported the development of tetrahydroisoquinoline (THIQ) 6-*O*-sulfamate-based compounds and evaluated their antiproliferative activity and ability to disrupt microtubule formation.^99^ Among the synthesized compounds, 42, featuring dichloro substituents on the benzyl ring, displayed the most potential anticancer activity across multiple cancer cell lines, with IC_50_ values as low as 90 nM (Fig. 17). It also displayed strong antitubulin activity, with an IC_50_ of 0.98 µM, making it the most effective tubulin inhibitor among the tested derivatives. Notably, the tubulin polymerization inhibition by 42 was only two-fold less potent than that of the clinically used microtubule disruptor combretastatin A-4 (CA-4). Additionally, 42 was found to bind the colchicine-binding site of tubulin, similar to CA-4. Literature comparisons highlighted 42 as the most promising THIQ derivative to date, showing 67% inhibition of tubulin polymerization at a concentration of 5 µM, confirming its potential as a lead compound for anticancer drug development.

**Fig. 18:**
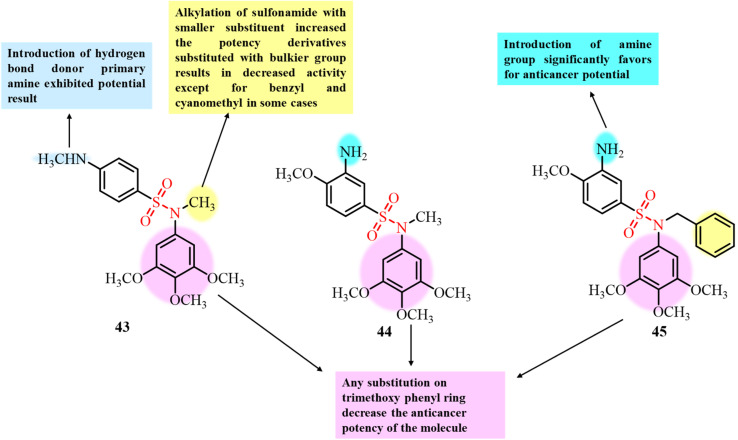
Chemical structures of developed sulfonamide-based derivatives 43–45 as tubulin polymerization inhibitors.

In 2021, González *et al.* reported the development of a series of 52 sulfonamide-based compounds as tubulin polymerization inhibitors binding at the colchicine site. The small molecular size of the designed compounds contributed to their favourable pharmacokinetic properties, enhanced aqueous solubility, and ability to evade the MDR efflux mechanism.^66^ Among the synthesized derivatives, compounds 43, 44, and 45 exhibited the most potent anticancer activity, with IC_50_ values in the nanomolar range against ovarian, cervical, and breast cancer cell lines, surpassing the efficacy of paclitaxel (Fig. 18). *In vitro* tubulin polymerization assays confirmed that these compounds significantly disrupted microtubule assembly in MCF-7, SKOV3, and HeLa cells. At a concentration of 20 µM, all three compounds achieved 50% inhibition of tubulin polymerization. Notably, compound 45, featuring a benzyl substituent on the sulfonamide group, displayed the strongest tubulin inhibition (IC_50_ = 3.7 µM), comparable to combretastatin A-4 (CA-4, IC_50_ = 3 µM). Compounds 43 and 44 showed moderate inhibition with IC_50_ values of 10 and 12 µM, respectively. Molecular docking further supported colchicine-site binding, consistent with CA-4. This study highlights sulfonamide-based scaffolds as promising antitubulin agents with potent antiproliferative potential.

**Fig. 19:**
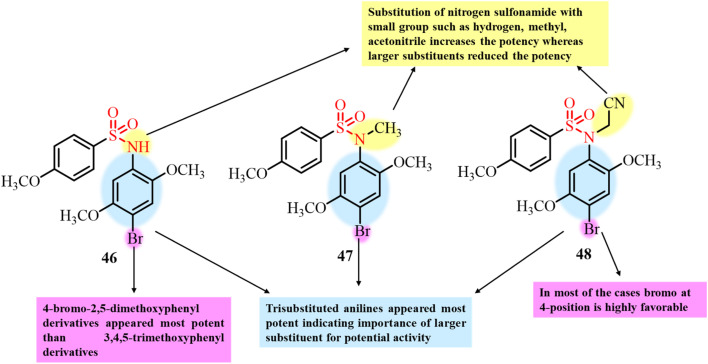
Chemical structures of 2,5-dimethoxyaniline–sulfonamide derivatives 46–48.

The same group in the same year reported the synthesis of another series of 37 compounds bearing sulfonamide functional groups.^100^ The compounds with 2,5-dimethoxyaniline, especially 4-brominated compounds 46–48, having IC_50_ values in the nanomolar range for HEK293 cells, were found to be the most potent compounds (Fig. 19). To investigate the target site of these compounds, they were screened at a concentration of 10 µM for tubulin polymerization inhibitory activity. Compound with most significant antiproliferative activity among the developed series also showed potential affinity to target microtubule polymerization with IC_50_ values less than 10 µM, indicating tubulin polymerization as potent target site for anticancer activity. Fluorescence imaging results further confirmed the disruption of the microtubule network and cellular mitotic arrest in MCF7 and HT-29 cells treated with compound 48. Molecular docking results also confirmed the binding of the ligand to the colchicine site in a different disposition, which was responsible for its high potency.

**Fig. 20:**
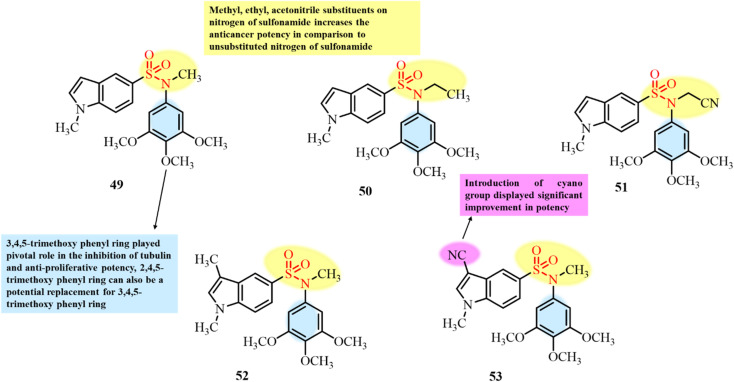
Chemical structures and SAR of indole–sulfonamide derivatives 49–53.

Vicente-Blázquez and colleagues (2025) reported the synthesis of a novel series of indole sulfonamide derivatives, featuring substituents at the indole 3-position, as potential colchicine-site binders and tubulin inhibitors for cancer therapy. These compounds displayed potent anticancer activity with IC_50_ values in the sub-micromolar to nanomolar range across four tumour cell lines.^101^ Among them, compounds 49, 50, 51, 52, and 53 showed the most significant anticancer effects, with structural modifications such as amide, formyl, and nitrile groups enhancing their drug-likeness (Fig. 20). *In vitro* and *in vivo* studies confirmed that the indole sulfonamides effectively inhibited tubulin polymerization, a key mechanism underlying their anticancer activity. Compounds 49 and 50 were particularly potent, with IC_50_ values of 2.1 µM and 2.2 µM, achieving 100% and 96% inhibition at 10 µM, respectively. Flow cytometry analysis of HeLa cells revealed early G2/M phase arrest, followed by increased cell death. Interestingly, while similar responses were observed in both cancerous and non-cancerous cells, apoptosis in cancer cells was rapid and irreversible, whereas in non-cancerous cells, it was delayed, which might be due to their differential cellular stress responses and the checkpoint integrity of tumor and non-tumor cells. Molecular docking confirmed colchicine-site binding, with key interactions in zones A and B of the tubulin interface.

**Fig. 21:**
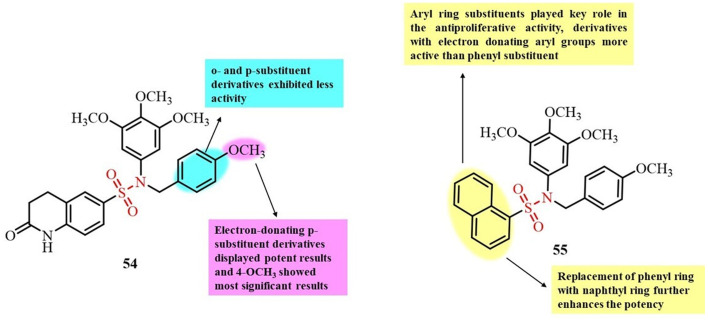
Chemical structures of quinolone-sulfonamide derivative 54 and naphthalene sulfonamide functional group derivative 55.

Ma *et al.*, in 2021, reported the development of a novel series of quinolone–sulfonamide derivatives (Fig. 21). The antiproliferative activities of all the synthesized derivatives were investigated against different cancer cell lines including HeLa, A549, HCT116, HePG2 and L02. Compound 54 showed excellent *in vitro* cytotoxicity results against all five tested cancer cell lines, with IC_50_ values less than 5 µM.^102^ Compound 54 was further analyzed to investigate its effect on tubulin polymerization inhibition. The results indicated that compound 54 significantly inhibited the tubulin polymerization assembly, with an IC_50_ value of 6.74 µM. Based on these results, it can be concluded that the potent antiproliferative activity of compound 54 is primarily due to its ability to disrupt microtubule formation. This mechanism was further supported by molecular docking studies, which aligned with experimental findings and confirmed the binding of compound 54 to the colchicine site of tubulin.

Wang and colleagues reported the synthesis of a novel series of sulfonamide derivatives containing a naphthalene moiety and evaluated their antiproliferative and tubulin polymerization inhibition potential (Fig. 21). These compounds were tested against the MCF-7 and A549 cancer cell lines, with compound 55, bearing a naphthalen-1-yl group, showing the most promising activity, exhibiting IC_50_ values of 0.51 µM (MCF-7) and 0.33 µM (A549).^103^ In tubulin polymerization assays, 55 demonstrated significant inhibitory activity with an IC_50_ of 2.8 µM, outperforming colchicine (IC_50_ = 9.3 µM). Additionally, compound 55 induced apoptosis *via* G2/M-phase cell cycle arrest in MCF-7 cells and exhibited low cytotoxicity toward normal human cell lines. Molecular docking studies further supported the mechanism of action, revealing that 55 binds at the colchicine-binding site of tubulin with a binding score of −9.6 kcal mol^−1^. The naphthalene ring was positioned in a hydrophobic pocket, engaging in strong hydrophobic interactions with the surrounding residues, while hydrogen bonding was observed between 55 and Lys254. These findings highlight that compound 55 is a promising lead for chemotherapeutic development *via* tubulin inhibition.

**Fig. 22:**
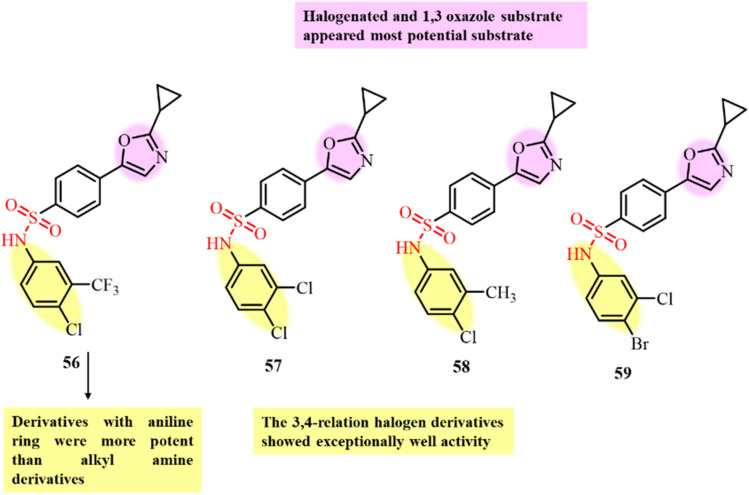
Chemical structures and SAR of oxazole sulfonamide derivatives 56–59.

Sisco *et al.*, in 2021, prepared a novel series of 1,3-oxazole sulfonamide derivatives and screened them for their potential to prevent cancer cell proliferation (Fig. 22). The developed compounds were screened against the NCI-60 human tumour cancer cell line.^104^ Most of the synthesized derivatives displayed significant potential growth inhibition activity. Especially, the aniline derivatives bearing halogen substituents showed the most promising results by inhibiting cancer cell growth by 75%. The most effective derivatives exhibited GI_50_ values in the sub-micromolar to nanomolar range and showed minimal overall toxicity, as observed by their high LC_50_ values. Further, *in silico* studies of compounds 56, 57, 58 and 59 suggested that they induce depolymerization by preventing self-polymerization and directly binding with tubulin. Compound 56 showed an IC_50_ value of 0.22 µM, while 57, 58 and 59 displayed more potent results with IC_50_ values of less than 0.08 µM.

**Fig. 23:**
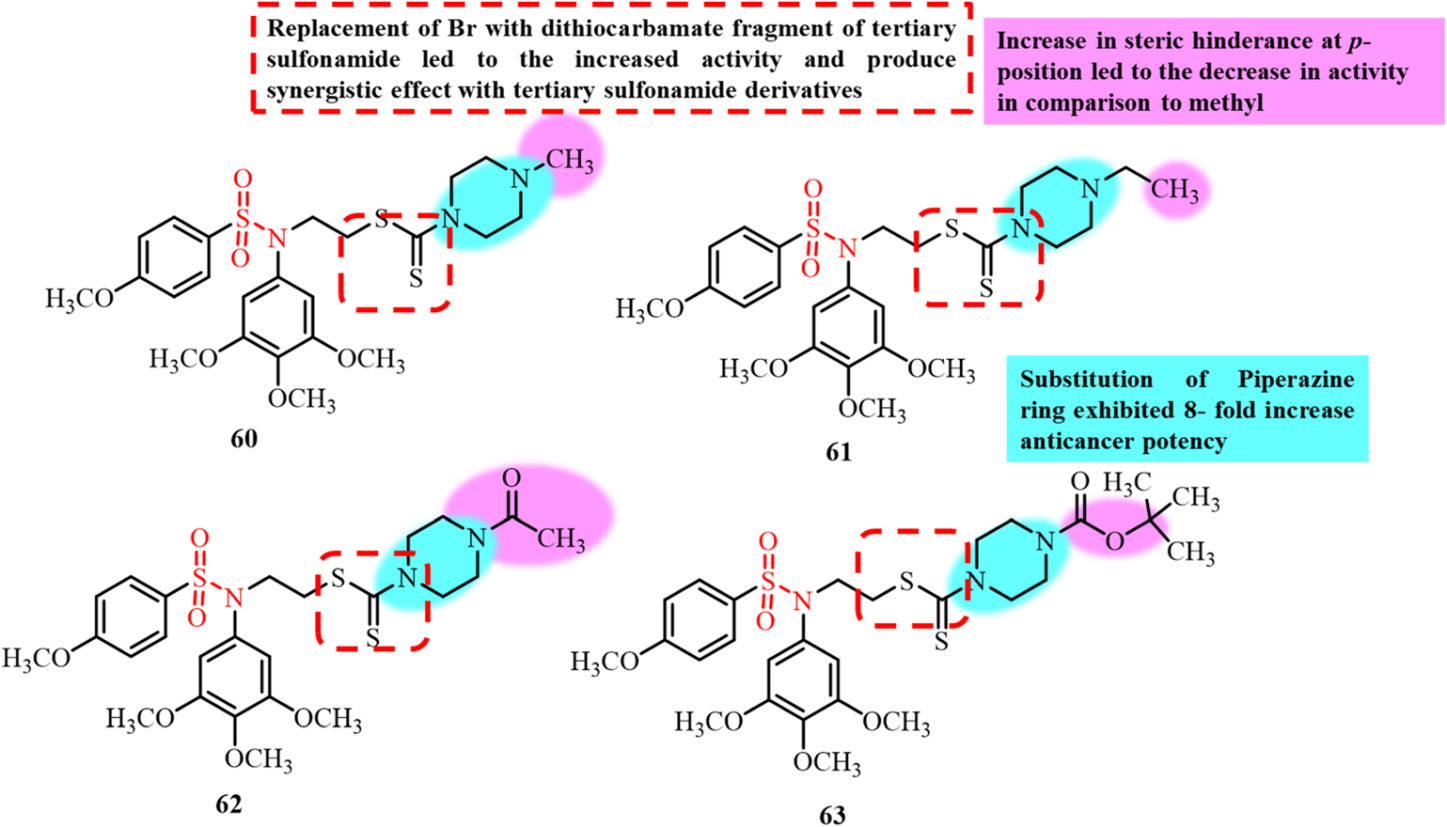
Chemical structures of tubulin polymerization inhibitor sulfonamide derivatives 60–63.

Ding *et al.*, in 2021, reported the synthesis of tertiary sulfonamide derivatives and investigated their antiproliferative activity against lung cancer cell lines (SNU-475, HepG2 and Bel-7402).^105^ These derivatives were developed as dual targets for lung cancer therapy *via* tubulin polymerization inhibition and lysine-specific demethylase 1 (LSD1). Among the tested compounds, 60 displayed the strongest antiproliferative activity against the Bel-7402 cancer cell line with an IC_50_ of 0.32 µM. An *in vitro* tubulin-polymerization assay revealed significant inhibition of tubulin polymerization by 60, 61, 62 and 63 with IC_50_ values of 1.27 µM, 2.13 µM, 1.78 µM and 3.07 µM, respectively (Fig. 23). The results showed that replacement of the methyl group with larger substituents on piperazine led to a decrease in activity. Immunofluorescence assays further confirmed that 60 and the other tested compounds significantly disrupted microtubule structures, with this effect being the most pronounced in derivatives 60 and 61. The best docking pose of compound 60 with tubulin (PDB structure) revealed key interactions at the colchicine-binding site. The 3,4,5-trimethoxyphenyl fragment and the tertiary sulfonamide moiety form two hydrogen bonds with Val353 and Ala247, respectively. Additionally, the dithiocarbamate group establishes a hydrogen bond interaction with Asn101. These interactions support the potential of 60 as a tubulin polymerization inhibitor.

**Fig. 24:**
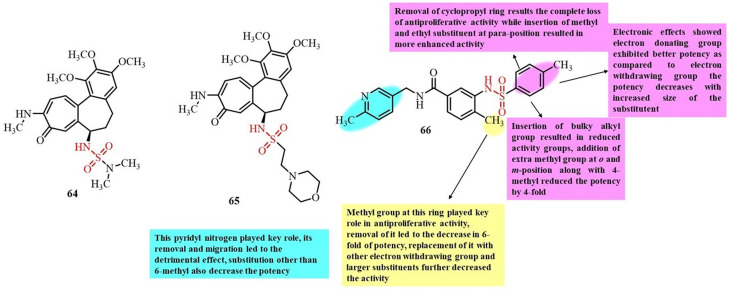
Chemical structures and SAR of colchicine-based sulfonamide derivatives 64 and 65 and sulfonamide-functionalized benzamide derivative 66.

Krzywik and colleagues (2020) developed a series of novel colchicine-based sulfonamide derivatives, doubly modified at the C7 (amide, sulfonamide, or sulfamide groups) and C10 (methylamino group) positions to enhance their anticancer activity, while minimizing toxicity (Fig. 24).^106^ The developed compounds were evaluated for their antiproliferative effects against A549, MCF7, LoVo, BALB/3T3, and drug-resistant LoVo/DX cancer cell lines. Among them, sulfonamide derivatives 64 and 65 exhibited the most promising results, with IC_50_ values in the 10–15 nM range across multiple cancer cell lines. Molecular docking studies supported their tubulin inhibition potential, with compound 64 displaying a strong binding score of −54.6 kcal mol^−1^. Compound 65, bearing a morpholine group, showed an even lower docking score (−59.6 kcal mol^−1^), suggesting its stronger theoretical binding, although it exhibited weaker antiproliferative activity, which is likely due to steric hindrance or efflux-mediated resistance. The difference in the results of *in vitro* biological activity and *in silico* computational studies was observed and it can be explained due to the several additional factors taking place in human body. Alternatively, in computational simulation, the focus was only on the binding mode of the compound with the target. The primary factor assumed for the difference in the results is off-target interactions involving efflux transporters with different affinities for the different compounds and differences in the solubility of the developed compounds. Also, differences in the expression of β-tubulin isotypes in different cancer cells also contribute to the different results from the two studies. Thus, these sulfonamide-modified colchicine derivatives represent potential drug candidates for further development as tubulin polymerization inhibitors capable of overcoming drug resistance in cancer therapy.

The study by Lin *et al.*, in 2022, presented the development of benzamide derivatives bearing a sulfonamide moiety as excellent tubulin inhibitors for anticancer activity (Fig. 24). Compound 66 having a methyl pyridyl substituent emerged as the most potent compound among them.^107^ Compound 66 displayed potential anticancer activity against several cancer cell lines in the nanomolar range including drug-resistant A549 and MCF-7R cells. *In vivo* studies further confirmed the promising results for the inhibition of tumor growth in prostate, lung, and melanoma tumor xenograft models treated with compound 66. Molecular docking studies revealed deep binding of 66 at all three sites of the colchicine domain on tubulin, and hence its potential to inhibit tubulin polymerization. An *in vitro* tubulin polymerization assay revealed disruption of the microtubule network in DU145 prostate cancer cells treated with 66 and induction of cell apoptosis by G2/M-phase cell cycle arrest. Thus, this study highlights the potential of 66 as a promising next-generation chemotherapeutic agent acting through tubulin polymerization inhibition.

**Fig. 25:**
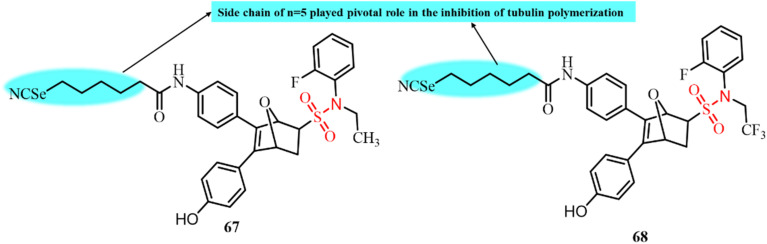
Chemical structures of novel selenocyanate-modified sulfonamide derivatives 67 and 68 as dual-target anticancer agents.

Deng *et al.* described the potential of novel selenocyanate-modified sulfonamide derivatives as dual targets for estrogen receptor α (ERα) degradation and tubulin polymerization inhibition (Fig. 25).^108^ They developed this series through the introduction of a selenocyanate-modified sulfonamide group in the bridged bicyclic scaffold. The developed compounds displayed potent ERα degradation and inhibition of tubulin polymerization. Compound 67 (IC_50_ = 0.09 µM) and 68 (IC_50_ = 0.06 µM) emerged as the most promising antiproliferative agents against MCF7 cancer cells, with potential of dual targeting for ERα degradation and tubulin polymerization inhibition. The tested compounds appeared less toxic with significant safety profiles for a normal breast cell line. Thus, the developed compounds have significant cancer cell selectivity with a good therapeutic window, as indicated by their potent SI values (>50). These compounds effectively disrupted chaotic nucleus, microtubule integrity and inhibition of microtubule polymerization in comparison to colchicine, causing G2/M phase cell cycle arrest. Their tubulin polymerization inhibition was even better than a known derivative. Thus, SeCN-modified sulfonamide derivatives are crucial for tubulin inhibition and increased pharmaceutical efficacy. Hence, this study provides a new strategy to overcome endocrine resistance and enhance antiproliferative efficacy *via* the combined degradation of ERα and inhibition of tubulin polymerization.

**Fig. 26:**
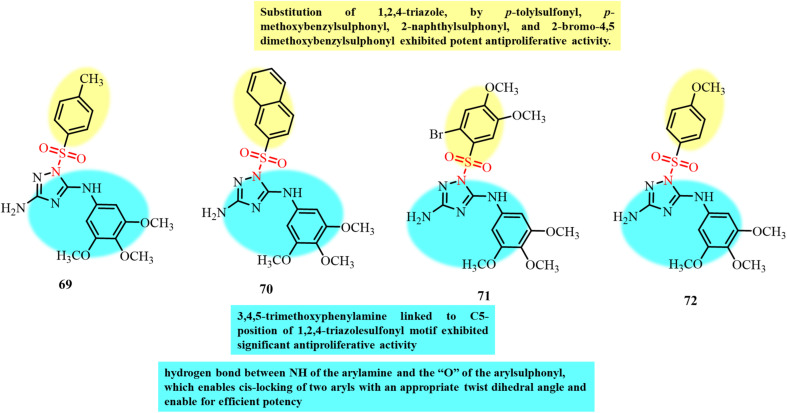
Chemical structures and SAR of trimethoxyphenyl-1,2,4-triazolosulfonamide derivatives 69–72.

Rao *et al.* developed a novel series of natural product-inspired trimethoxyphenyl-1,2,4-triazolosulfonamide derivatives as potential tubulin polymerization inhibitors (Fig. 26). The designed compounds were developed through a rational medicinal chemistry approach and screened for their antiproliferative activity against the MCF-7 cancer cell line.^109^ Among them, compounds 69, 70, 71, and 72 showed significant growth inhibition (60–73%) at a concentration of 5 µM, with compound 70 emerging as the most potent candidate. Tubulin polymerization assays revealed the significant interaction of compound 70 with the colchicine site of tubulin, effectively suppressing tubulin polymerization and depolymerizing microtubules both *in vitro* and in cancer cells. Its inhibition rates were 9%, 19%, and 39% at 1, 2, and 5 µM, respectively, with a noticeable reduction in microtubule density at higher concentrations. A dose-dependent decrease in the fluorescence intensity of the colchicine–tubulin complex further confirmed the strong binding of compound 70 at the colchicine-binding site, outperforming the other derivatives. Biological assays showed that compound 70 induced G2/M-phase arrest, multi-nucleation, mitotic slippage, and polyploidy. SAR analysis highlighted the importance of *cis*-locked aryl groups, a trimethoxyphenyl substituent at the C5 position, and sulfonyl-linked hydrogen bonding for its activity. Compound 70 exhibited favourable pharmacological properties and holds strong promise as a tubulin-targeting anticancer agent.

**Fig. 27:**
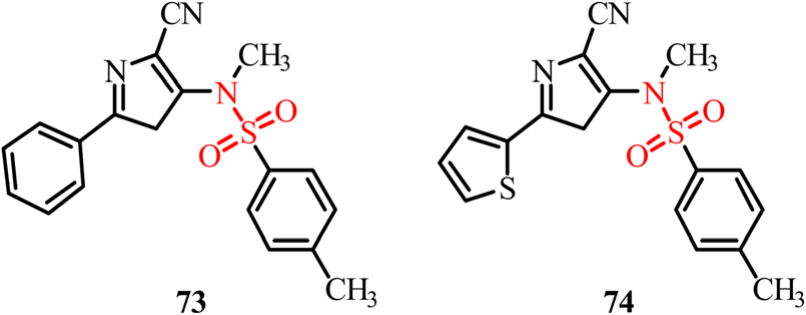
Chemical structures of *N*-(4-cyano-1,3-oxazol-5-yl)sulfonamide derivatives: 73 and 74.

Severin *et al.* reported the development of 14 *N*-(4-cyano-1,3-oxazol-5-yl)sulfonamide-based derivatives and evaluated their antiproliferative activity against the NCI-60 cancer cell line panel (Fig. 27). Among them, compounds 73 and 74, bearing 4-phenyl and thiophene substituents, respectively, displayed the most promising anticancer activity, particularly against leukemia and breast cancer cell lines with growth inhibition of more than 50% in all the tested cell lines.^110^ Five-dose assays confirmed their inhibitory activity with GI_50_ values in the micromolar range, though their cytotoxic concentrations exceeded 100 µM, suggesting primarily cytostatic effects. COMPARE analysis indicated that disruption of microtubule network formation might be a possible mechanism responsible for their significant anticancer activity. Therefore, these derivatives can be used for further modification to develop more effective chemotherapeutic agents in the future.

**Fig. 28:**
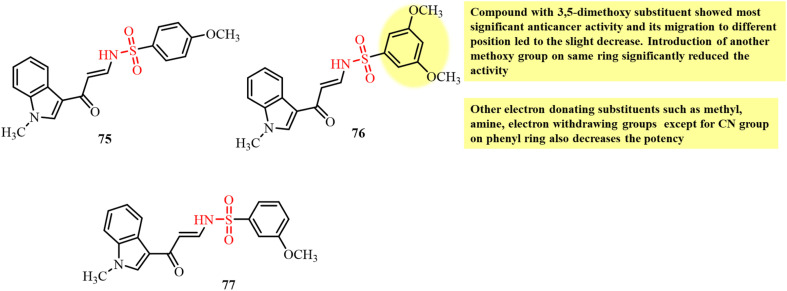
Chemical structures of indolylaryl sulfonamide conjugates 75–77 as tubulin polymerization inhibitors.

Dasari *et al.* developed a novel series of indolylaryl sulfonamide conjugates as tubulin polymerization inhibitors having potential antiproliferative activity (Fig. 28). The compounds were screened against A549, HeLa and MCF7 cancer cell lines to investigate their antiproliferative activity, where nocodazole was used as the standard reference drug.^111^ Compounds 75, 76 and 77 substituted with 4-methoxy-, 3,5-dimethoxy- and 3-methoxy-phenyl rings, respectively, emerged as the most potent antiproliferative agents among them, with IC_50_ values of ≤2 µM against all the tested cancer cell lines. The molecular docking pose of 75 with α,β-tubulin showed strong interactions with a binding score of −8.11 kcal mol^−1^. An *in vitro* tubulin polymerization assay revealed the potent inhibitory activity of the tested compounds with IC_50_ values in the range of 2.34 µM to 3.23 µM, and the results were comparable to the known tubulin inhibitor combretastatin A-4. Thus, these developed indole-aryl sulfonamide conjugates appear to be promising scaffolds for the further design and development of tubulin polymerization inhibitors with potent anticancer activity.

## 5. Conclusion and future perspectives

The discovery of microtubule-targeting agents (MTAs) marks a pivotal milestone in anticancer drug development. These agents disrupt one of the most critical processes in cell division, tubulin polymerization, placing them at the forefront of cancer therapeutics. Among MTAs, sulfonamide derivatives have emerged as highly versatile and privileged scaffolds due to their structural flexibility and strong affinity for the colchicine-binding site on tubulin. Their mechanism of action typically involves disrupting microtubule dynamics, inducing G2/M-phase cell cycle arrest, and triggering apoptosis in rapidly dividing cancer cells.

This review highlights the strong antiproliferative activity of sulfonamide-based tubulin inhibitors across a broad range of cancer cell lines, including multidrug-resistant phenotypes. These compounds also display favourable pharmacokinetic and pharmacodynamic (PK/PD) profiles, which can be optimized through rational chemical modifications. Importantly, many sulfonamide derivatives show reduced susceptibility to P-glycoprotein-mediated drug efflux, addressing a key limitation of conventional MTAs such as taxanes and vinca alkaloids.

Advancements in molecular hybridization, SAR studies, and *in silico* docking have enabled the development of dual- or multi-target sulfonamide agents, which are capable of simultaneously inhibiting tubulin and oncogenic proteins such as STAT3 or ERα. These multifunctional compounds offer improved efficacy with reduced resistance and off-target effects. Extensive structure–activity relationship (SAR) investigations demonstrate that rational modification of distinct molecular moieties plays a pivotal role in compound optimization. The potent antiproliferative activity of sulfonamide derivatives as tubulin inhibitors is strongly influenced by their structural and physicochemical characteristics. A critical feature is their conformational flexibility, which enables optimal accommodation within the colchicine-binding site of β-tubulin. In particular, the sulfonamide moiety can participate in key hydrogen-bonding interactions with essential amino acid residues such as Cys251 and Ala250, thereby stabilizing ligand–protein binding and enhancing inhibitory activity. The presence of electron-rich aromatic systems, especially methoxy-substituted phenyl rings, and more notably the trimethoxyphenyl motif, plays a crucial role in strengthening interactions within the colchicine-binding pocket. These pharmacophores enhance π–π stacking and hydrophobic interactions, facilitating improved binding affinity and microtubule polymerization inhibition.

Incorporation of heterocyclic scaffolds such as indole, quinoline, carbazole, and triazole has further been shown to enhance antiproliferative activity. These heterocycles contribute to increased lipophilicity and improved cellular permeability, enabling more efficient intracellular accumulation. In certain cases, they also allow engagement with secondary molecular targets, thereby introducing a dual-target mechanism that can enhance anticancer efficacy, particularly in drug-resistant cancer cell lines. Additionally, the introduction of functional extensions such as amide, carbonyl, or thiocarbamate linkages has been associated with improved biological activity. These groups may optimize hydrogen-bonding capacity, modulate electronic distribution, and favorably influence pharmacokinetic properties, including metabolic stability and bioavailability.

Overall, the antiproliferative efficacy and tubulin inhibitory potential of sulfonamide derivatives depend on a combination of key binding interactions within the colchicine site and strategic structural modifications that optimize their pharmacodynamic and pharmacokinetic profiles. Careful tuning of these molecular features is essential for the development of sulfonamide-based tubulin inhibitors as clinically effective and safer anticancer therapeutics. The key interactions of the reported molecules of this review are summarized in Table 6.

**Table 6:** Important interactions reported between sulfonamide derivatives and amino acid residues

Compound	Amino acid	Interactions present
1	Cys241 + other amino acids	H-bond + van der Waals forces + hydrophobic
3	Ala-354, Leu-248, Cys-241, Val-318 and Lys-352 + Ala-316	Pi-alkyl interaction + sigma interaction
4	Val 238	H-bond
12 and 13	Cys241 + Leu255, Ala250, Cys241, Ala354, Lys352 + Met259	H-bond + Pi-sigma interaction + Pi-alkyl and alkyl bonds + Pi-σ
14	Asn258, Cys241 + Leu255, Leu248 + Leu255, Leu242, Val238, Leu242, Ala250, Ala354, Cys241	H-bond + Pi-σ, Pi-alkyl and alkyl interaction
16	Cys241 + Leu255, Leu248 + Cys241, Leu255, Leu242, Ala250, Lys352, Met259, Ala354	H-bond + Pi-σ + Pi-alkyl and alkyl interactions
17	Leu255, Asn258, Cys241 + Leu255	H-bond + Pi-σ + Pi-alkyl and alkyl bonds + Leu255, Ala250, Cys241, Ala250, Lys352
21	Gln11 + Asn258	H-bond
22	Gln11	H-bond
25	Tyr202, Asp251 + Ala 180, Val 181 and β-chain residues such as Leu248, Ala250, Leu252, Leu255, and Met259	H-bond + hydrophobic interactions
26	βAsn258, βMet259 and βLys352 + αThr179	Hydrophobic interaction + H-bond
27 + 28	Val238, Thr179, Thr353 + Leu248, Leu255, Cys241, Lys352, and Ala250	Hydrogen bond + hydrophobic interactions
30, 31, 32 and 34	Asn258β + Met259β, Thr314β, and Lys352 β + Thr179α	Pi-interaction + hydrophobic interactions + H-bonding
ELR510444	N256 + C239, L240, L246, A248, L253, A314, K350, and A352	H-bonding + hydrophobic interaction
35	N165, E198, V236 + C239, L246, L253, M257, A314, K350, A352, and I368	H-bonding + hydrophobic interaction
37	Cys241, Val238, Lys254, Asn101 and Ser178 + Ala250, Ala180, Leu248, Ala316, Ile378 and Leu255	H-bonding + hydrophobic interactions
40	Ala316, Lys352 and Ser178	H-bonding
41	Val101, Leu103, Val486, His485, PHE488 + THR165, Lys134, Asp36 and Lys368	Van der Waals interaction + H-bonding
54	Asn167, Val238 + Thr179, Ser178 + Leu255, Ala250, Ala316, Cys241, Ile378, Ala354, Lys352	H-bond + C–H bond + Pi-alkyl + van der Waals interaction
55	Ala-180, Val-181, Val-238, Leu-248, Ala-250, Leu-255, Ala-316, Ala-317, Val-318 and Ala-354 + Lys-25	Hydrophobic binding + cation-pi interaction + H-bonding
60	V353, A247, N101 + W346, P348 and F343	H-bonding + hydrophobic interaction
64	Gln10, Gly142, Gly143, Thr144 and Ser177	α-tubulin
65	Asp68, Asn100 + Lys687	α-tubulin + β-tubulin
66	Leu253, Asn256, Cys 239, Tyr200	H-bond
67	Gly144, Asp251	H-bond
68	Gly144, Glu183, Ser140, Asp179	H-bond
Colchicine	Valα181, Serα178, and Valβ315 + Val181α, Cysβ241	Van der Waals interaction + H-bonding

Preclinical studies further support the therapeutic promise of sulfonamide derivatives, demonstrating significant tumor inhibition *in vivo* with minimal toxicity to normal cells. Going forward, integrating high-resolution structural biology, computational modeling, and medicinal chemistry will be essential for developing the next generation of sulfonamide-based MTAs. Priorities include enhancing tumor selectivity, solubility, metabolic stability, and employing targeted delivery systems to improve therapeutic outcomes.

In summary, sulfonamide derivatives represent a powerful and adaptable class of anticancer agents targeting tubulin polymerization. Their effectiveness, resistance–avoidance potential, and amenability to structural optimization make them strong candidates for future chemotherapeutic development. Continued interdisciplinary research will be key to advancing these compounds into clinical trials and providing safer, more effective cancer treatments (Table 7).

**Table 7:** Structural features of tubulin-targeting drug candidates for addressing drug resistance

S. no.	Structural feature	Effect on activity	Resistance-type addressed	Example	Ref.
1	Trimethoxy phenyl, and pentafluoro-phenyl ring substitution	Tubulin affinity, π-interaction and hydrophobic interaction increase	Multidrug	T138067	112
2	Introduction of heterocyclic ring	Colchicine domain tubulin inhibitor and increased anticancer activity	Overcomes glycoprotein and βIII isoform overexpression-mediated resistance	ELR510444, ABT-751	25 and 113

The potential structural flexibility and modifiable pharmacological properties of sulfonamide derivatives make them significant scaffolds for designing and developing next-generation anticancer drugs. Future research should focus on rational hybrid design, resistance-evasion strategies, and dual-targeting strategies to overcome the shortcomings of existing therapeutics. Co-developing precise modeling with the pre-clinical verification will also fast-track the translation of sulfonamide-based tubulin inhibitors into viable, clinical anti-cancer agents.

## Conflicts of interest

The authors declare no competing financial interest.

## Data Availability

No primary research results, software or code have been included and no new data were generated or analysed as part of this review.
